# Highly Stretchable Conductive Covalent Coacervate Gels for Electronic Skin

**DOI:** 10.1021/acs.biomac.1c01660

**Published:** 2022-02-21

**Authors:** Nam T. Nguyen, James Jennings, Amir H. Milani, Chiara D. S. Martino, Linh T. B. Nguyen, Shanglin Wu, Muhamad Z. Mokhtar, Jennifer M. Saunders, Julien E. Gautrot, Steven P. Armes, Brian R. Saunders

**Affiliations:** † Department of Materials, University of Manchester, MSS Tower, Manchester M13 9PL, U.K.; ‡ Department of Chemistry, University of Sheffield, Sheffield S3 7HF, U.K.; § School of Engineering and Materials Science, Queen Mary University of London, London E1 4NS, U.K.; ∥ Eastman Dental Institute, University College London, London WC1X 8LD, U.K.

## Abstract

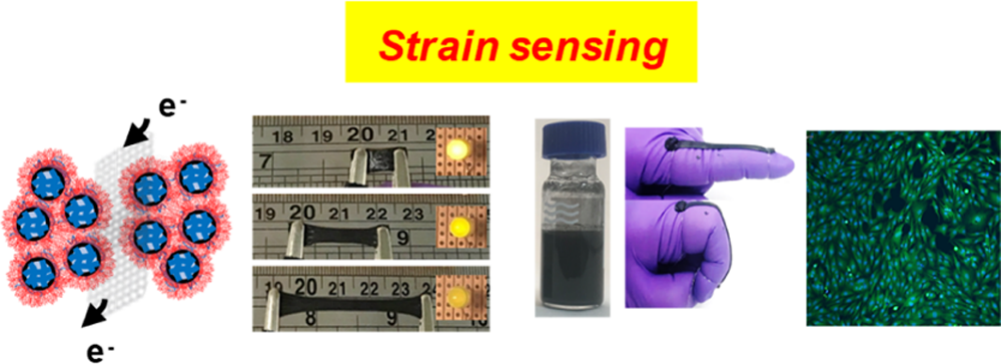

Highly stretchable electrically conductive hydrogels have been extensively researched in recent years, especially for applications in strain and pressure sensing, electronic skin, and implantable bioelectronic devices. Herein, we present a new cross-linked complex coacervate approach to prepare conductive hydrogels that are both highly stretchable and compressive. The gels involve a complex coacervate between carboxylated nanogels and branched poly(ethylene imine), whereby the latter is covalently cross-linked by poly(ethylene glycol) diglycidyl ether (PEGDGE). Inclusion of graphene nanoplatelets (Gnp) provides electrical conductivity as well as tensile and compressive strain-sensing capability to the hydrogels. We demonstrate that judicious selection of the molecular weight of the PEGDGE cross-linker enables the mechanical properties of these hydrogels to be tuned. Indeed, the gels prepared with a PEGDGE molecular weight of 6000 g/mol defy the general rule that toughness decreases as strength increases. The conductive hydrogels achieve a compressive strength of 25 MPa and a stretchability of up to 1500%. These new gels are both adhesive and conformal. They provide a self-healable electronic circuit, respond rapidly to human motion, and can act as strain-dependent sensors while exhibiting low cytotoxicity. Our new approach to conductive gel preparation is efficient, involves only preformed components, and is scalable.

## Introduction

Hydrogels are polymer networks containing water. Increasingly sophisticated understanding of their structure–property relationships has transformed them from relatively weak, brittle materials to tough,^1,2^ resilient materials with mechanical properties comparable to those of rubber.^3−5^ Of particular success has been the introduction of multiple networks within hydrogels, including sacrificial dense networks for efficient energy dissipation.^4,6,7^ The resulting improvement in mechanical properties suggests new potential applications such as cartilage augmentation^8^ and soft robotics.^9^ Inclusion of electrically conductive additives to such gels has potential to confer conductivity^10,11^ with minimal reduction in mechanical properties.^12−14^ This has enabled electronic skin applications to be explored.^15−19^ The most common method to prepare high-performance hydrogels involves in situ free-radical polymerization.^14,20,21^ This approach is attractive because the comonomers can rapidly diffuse into pre-existing networks.^22^ In principle, alternative approaches involve linking together polymeric precursors to form a gel using epoxy-amine chemistry, Schiff base reactions,^23^ host–guest interactions,^24^ and/or noncovalent interactions between the various components.

**Scheme 1:**
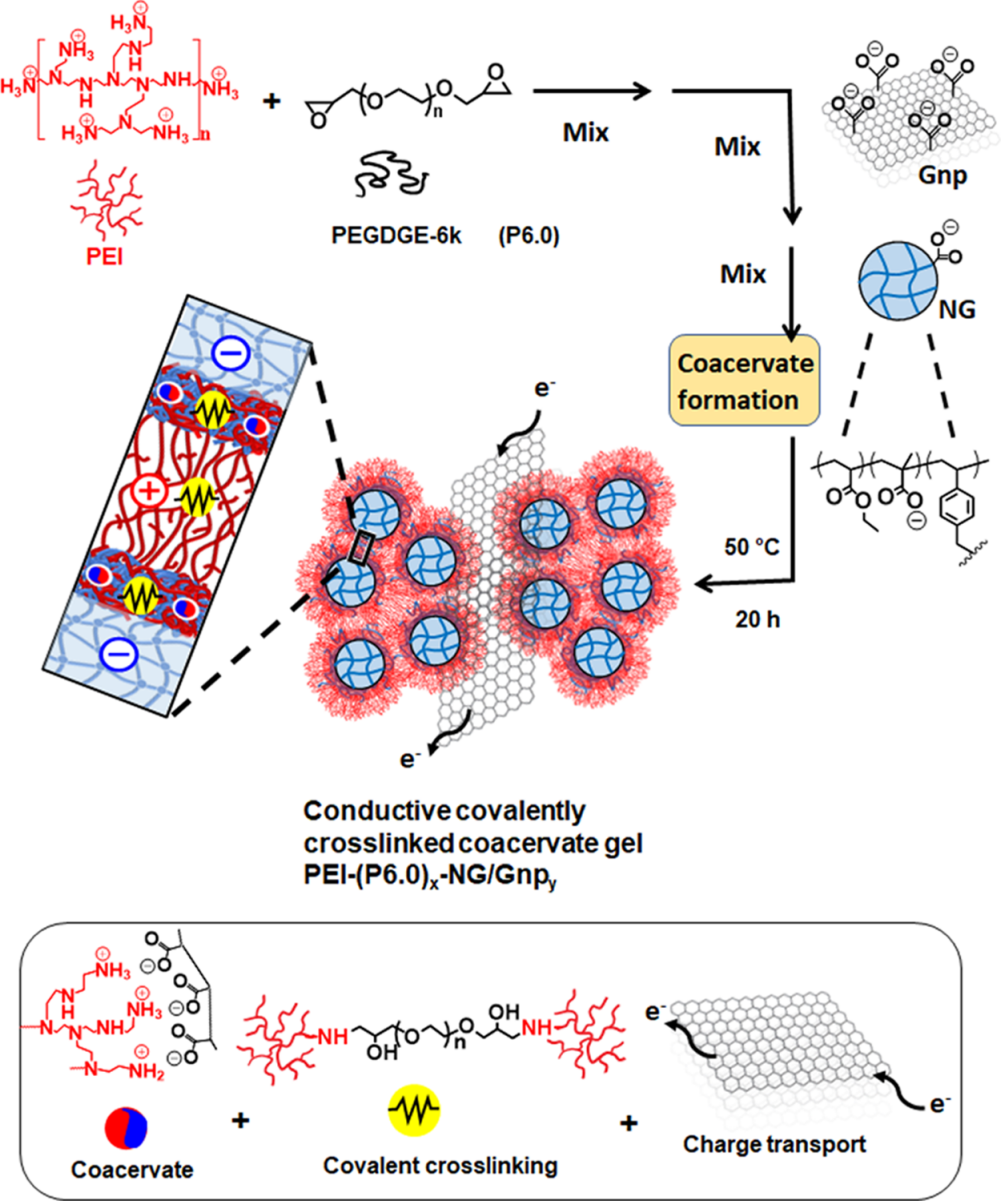
Depiction of the Preparation of Conductive Covalently Linked Complex Coacervate Gels These new gels combine coacervate formation, covalent cross-linking, and charge transport; the main focus of the study is PEI-(P6.0)_*x*_-NG/Gnp_*y*_ gels, where the values of *x* and *y* are the concentrations of PEGDGE and graphene nanoplatelets used during preparation in wt %.

In this study, we investigate gels prepared by a covalently linked coacervate approach using nanogels (NGs), polyethyleneimine (PEI), and polymeric di-epoxide as a tri-component system.^25−28^ NGs are sub-100 nm cross-linked polymer particles that swell when the pH exceeds the particle p*K*_a_.^29^ The coacervate in this study forms due to the ionic bonding of oppositely charged PEI and NG particles (see Scheme 1). This process occurs very rapidly when the two oppositely charged components are mixed and results in syneresis and an increase in the concentration of the components. Here, we use the di-epoxide polymer [poly(ethylene glycol) diglycidyl ether, PEGDGE] to covalently link PEI and show that this causes a dramatic improvement in the mechanical properties. We then include electrically conducting graphene nanoplatelets (Gnps) to provide an electrically conducting hydrogel with potential for electronic skin application.

Many studies have reported the construction of tough hydrogels,^3,30−32^ and double-network hydrogels are a well-known example.^3,33,34^ Wang et al. reported the formation of tough, strong alginate gels via water evaporation.^30^ However, the strain-at-break values were limited to ∼450% for the resulting ionically cross-linked gels. Truong et al. used a simultaneous orthogonal dual-click approach to produce tough hydrogels with breaking strains of ∼580% using norbornene-tetrazine and thiol-yne addition reactions.^31^ Murakami et al. prepared orthogonal double click reactions to prepare strong interpenetrating PEG-based gels with tensile yield strains of up to ∼800%.^32^ In this latter study, the single-network gels based on the reaction between epoxide- and amine-functionalized PEG precursors exhibited a strain of ∼1700%. In a separate study, PEGDGE was used to produce stretchable gels with a breaking strain of 230% via the ring-opening reaction of the epoxide with carboxylic acid groups.^25^

Introducing conductive additives within stretchable gels enables the design of strain sensors.^12−14,20^ Moreover, such additives can maintain^21^ or even enhance the mechanical properties of the gel.^14,35^ For example, carbon nanotubes have been added to gels to confer electrical conductivity.^5,21,36,37^ Conductive gels containing reduced graphene oxide have also been reported.^38^ Herein, we employ graphene nanoplatelets (Gnps) as the electrically conductive species. These nanoparticles are a relatively new form of graphene and consist of polycarboxylate-functionalized hydrophilic graphene nanoplatelets.^39^ They have been used to produce nacre-like materials,^40^ electro-responsive materials,^41^ and cathodes for solar cells.^42^ However, to the best of our knowledge, Gnps have not yet been used for electrically conductive gels.

The covalently cross-linked complex coacervate approach used for the first time in this study is designed to combine three attributes within one gel. First, we aim to improve a noncovalent complex coacervate approach recently developed by our group^43^ that involves combining PEI with anionic poly(ethyl acrylate-*co*-methacrylic acid-*co*-divinylbenzene) NG particles. Second, we use PEGDGE to covalently link the PEI chains. This is also intended to decrease the cytotoxicity of the gels. Third, we include Gnps to provide electrical conductivity. We show that judicious selection of the PEGDGE molecular weight also increases the stretchability and defies the toughness-strength paradox to give mechanical properties that are a rarity for most gels.^44,45^

We first investigate the effect of varying the PEGDGE molecular weight from 500 (P0.5) to 6000 (P6.0) g mol^–1^ on the mechanical properties of the PEI-(P0.5)_*x*_-NG and PEI-(P6.0)_*x*_-NG coacervate gels. We then incorporate Gnps to produce electrically conductive PEI-(P6.0)_*x*_-NG/Gnp_*y*_ gels and investigate the mechanical properties as well as potential use as strain sensors for both compression and tension. We demonstrate that the gels are self-adhesive and noncytotoxic and can detect rapid finger movements. This study demonstrates that it is straightforward to prepare highly stretchable conformal conductive strain sensors using a covalently linked complex coacervate. The compositions of the NGs can be readily varied, which implies that there is a wide range of cytocompatible compositions available for this new family of conductive gels.

## Experimental Details

### Materials

Ethyl acrylate (EA, 99%), methacrylic acid (MAA, 98%), divinyl benzene (DVB, 80%), ammonium persulfate (APS, 98%), sodium dodecyl sulfate (SDS, 98.5%), PEGDGE (99%) with molecular weights of 500 g/mol (P0.5) or 6,000 g/mol (P6.0), Gnps (polycarboxylate-functionalized, hydrophilic), and NaOH (99%) were purchased from Sigma-Aldrich (UK). PEI (branched, MW 10,000, 30 wt % aqueous solution) was purchased from Polysciences (Europe). Deionized water with a resistivity of 15 MΩ cm was produced from an SLS Lab Pro PURA-Q2 water purifier. Dulbecco’s modified Essential medium (DMEM), Hoechst 33342, live/dead viability/cytotoxicity kit containing ethidium homodimer-I, and calcein-AM were purchased from Thermo Fisher (UK). This kit was used according to manufacturer’s instructions. Fetal bovine serum (FBS), glutamine, penicillin, and streptomycin were purchased from Gibco, Invitrogen (UK). All chemicals were used as received.

### NG Synthesis

The dispersion of [poly(EA-*co*-MAA-*co*-DVB] NGs was synthesized via semicontinuous seed-feed aqueous emulsion polymerization, as reported elsewhere.^43^ Briefly, a comonomer solution containing EA (165.0 g, 1.65 mol), MAA (81.75 g, 0.95 mol), and DVB (1.30 g, 10.0 mmol) was prepared. SDS (1.8 g, 6.2 mmol) was dissolved in water (518 mL), and the solution was placed in the reaction flask and purged with nitrogen for 30 min at 80 °C. Part of the comonomer solution (31.5 g) was quickly added to the reaction flask via a funnel, and the solution was stirred for another 10 min under nitrogen. Aqueous K_2_HPO_4_ solution (3.15 mL, 7.0 wt %) was then added via a syringe and stirred for 2 min, followed by the addition of an aqueous APS solution (10 mL, 2.0 wt %). The copolymerization was allowed to proceed for 30 min at 80 °C. The remaining comonomer solution was then injected over a 90 min period using a syringe pump at a feed rate of 2.40 mL min^–1^. The copolymerization was stirred for a further 60 min after the feed was finished and then quenched in an ice bath. The resulting copolymer dispersion was purified by dialysis against water with continuous stirring for 7 days, during which water was replaced daily. The total solid content after dialysis was ∼4.0 wt %. The product was concentrated to 20 wt % for use in the gel preparations described below by room-temperature rotary evaporation.

### Synthesis of PEI-NG

PEI solution (0.80 g of 20 wt %) was added to an NG dispersion (1.40 g of 20 wt %), and the mixture was quickly stirred mechanically to form the complex coacervate pregel. Some syneresis was observed. The pregel was gently kneaded by hand for 2 min until it became smooth and uniform. (A video of this process has been published elsewhere.^46^) The gel pH was 9.8. The pregel was then transferred to an O-ring and sealed using nonadhesive polytetrafluoroethylene (PTFE) glass-fiber cloth, parafilm, and two glass slides secured with two clips. This gel was cured for 20 h in a 50 °C oven. The PTFE cloth ensured that the gel could be removed without any adhesion issues. Parafilm and glass slides were used to ensure that the samples were well sealed to prevent water evaporation.

### Synthesis of PEI-(P0.5)_*x*_-NG and PEI-(P6.0)_*x*_-NG

PEI-(P0.5)_*x*_-NG and PEI-(P6.0)_*x*_-NG were prepared using the same method. For PEI-(P6.0)_0.9_-NG, P6.0 (20 mg) was slowly added to an aqueous PEI solution (0.80 g of 20 wt %), while the vial was subjected to continuous vortex mixing. The mixture was then added to an NG dispersion (1.40 g of 20 wt %). The NG/PEI/P6.0 mixture had a pH of 9.6 and was stirred mechanically to form the coacervate. The pregel that formed was then treated in the same way as the PEI-NG, but in this case, it formed a covalent complex coacervate gel.

### Synthesis of PEI-(P6.0)_0.9_-NG/Gnp_*y*_ Gels

The protocol for the preparation of these gels is similar to that used for the synthesis of the PEI-(P6.0)_*x*_-NGs. To prepare PEI-(P6.0)_0.9_-NG/Gnp_3.1_ gel, P6.0 (20 mg) was slowly added to a 20 wt % aqueous PEI solution (0.80 g), while the vial was subjected to a continuous vortex. The freshly prepared PEI/P6.0 mixture was then transferred to a vial containing Gnps (70 mg) via pipette. The PEI/P6.0/Gnp mixture (pH 9.3) was vortex-mixed for 10 s and then sonicated at room temperature for 2 min. The mixture was then added to a 20 wt % NG dispersion (1.40 g) via a pipette. The mixture was then immediately stirred mechanically to form the coacervate. The treatment followed at this point is the same as that described above for the PEI-NG.

### Physical Measurements

Potentiometric titration data were obtained using a Mettler Toledo titrator and aqueous NaOH solution (1.0 M) as the titrant. An NG dispersion (0.50 wt %, 40 mL) was prepared using an aqueous solution of NaCl (0.050 M). Dynamic light scattering (DLS) and zeta potential data were obtained using a Malvern Zetasizer NanoZS instrument. The latter instrument used the CONTIN algorithm, and five replicate measurements were conducted. The scattering angle and temperature used were 173 and 25 °C, respectively. The particle concentration used for DLS and zeta potential measurements was 0.10 wt %, and the medium was phosphate or carbonate buffer (0.10 M). The latter buffers were used to vary the pH. The incident irradiation was via a HeNe laser (20 mW, 633 nm). The samples were not filtered. The viscosity and refractive index used were those for water and polystyrene, respectively. Malvern disposable cuvettes and universal dip cells were used for the DLS and zeta potential measurements, respectively. A Nicolet 5700 spectrometer (ThermoElectron Corporation) was used for Fourier-transform infrared (FT-IR) spectroscopy studies. Carbon-coated copper grids were used for the transmission electron microscopy (TEM) studies, and samples were stained using uranyl acetate solution (0.50 wt %). Imaging was performed at 100 kV using an FEI Tecnai 12 BioTwin instrument. The concentration used for depositing particles for scanning electron microscopy (SEM) and TEM investigation was 0.010 wt %. Number-average diameters were determined using Image-J (NIH) software. For SEM measurements, the hydrogels were rapidly frozen in liquid nitrogen and then freeze-dried overnight. The samples were mounted on Al slides using carbon tape and coated with Au. A Philips field-emission gun–SEM instrument operating at an accelerating voltage of 12 kV was used.

All uniaxial compression tests were performed using an Instron 3344 instrument. The gels were prepared in PTFE cylindrical molds with typical dimensions of 11.50 mm height and 11.00 mm diameter. Gels were compressed between two plates with a strain rate of 2.0 mm min^–1^ until either fracture was observed or the maximum load value was reached. The engineering stress is used in these studies. Rectangular gels (typically length = 18 mm, width = 6.5 mm, and thickness = 2.5 mm) were used for tensile tests. Gel samples were clamped and studied using a strain rate of 4.0 mm min^–1^. For lap-shear experiments, cylindrical gels with typical dimensions of 18 mm diameter × 2.5 mm thickness were placed between two PMMA substrates, which were then subjected to a 500 N load for 5 s prior to the experiment. Measurements were conducted using a strain rate of 4.0 mm min^–1^. Substrates were prepared in-house with the following dimensions: 76 mm length, 25 mm width, and 6.5 mm thickness.

### Small-Angle X-ray Scattering Experiments

Small-angle X-ray scattering (SAXS) measurements were performed in the SMALL laboratory at the University of Sheffield using a Xeuss 2.0 instrument (Xenocs, Sassenage, France) at room temperature and pressure. X-rays (λ = 1.341 Å) were generated using a liquid gallium MetalJet X-ray source (Excillum, Kista, Sweden) and collimated using two scatterless slits (1.2 and 0.8 mm). Two-dimensional scattering patterns were recorded using a Pilatus 1 M area detector at a sample-to-detector distance of 2.5 m (calibrated with silver behenate) and azimuthally integrated within the Foxtrot software package to reduce the data to one-dimensional scattering profiles. Liquid samples were analyzed in 2 mm borosilicate capillaries for 3 × 300 s exposures, while gel samples were analyzed using an array stage for 3 × 300 s exposures. The scattering vector (*q*) values for structure factor maxima (*q*_max_) were estimated to obtain average NG center-to-center distances (*D*) using the relationship *D* = 2π/*q*_max_.

### Electrical Measurements

All electrical measurements were performed using a Keithley 2701E multimeter and controlled using Kickstart software. The hydrogels were fabricated in O-rings and were cut into rectangular shapes (typically length = 18 mm, width = 6.5 mm, and thickness = 2.5 mm). In strain-sensing experiments, the hydrogel resistance was measured in real time during either compression or stretching of the gel. Both ends of the gel were clamped to a copper strip with an adhesive layer on one side. A copper wire connected each end of the copper strip to the Keithley 2710E. Resistance measurements were performed using a two-wire setup. Finger movement-sensing measurements were carried out by measuring the real-time resistance using a rectangular gel prepared as described above. This gel was placed on the forefinger of a nitrile glove. Two wires were used to form circular rings surrounding the finger at opposite ends of the rectangular gel.

### Cell Viability Studies

An HCA2 human fibroblast cell line was cultured in DMEM supplemented with 10% FBS, 1% glutamine, and 1% penicillin and streptomycin in a humidified 5% CO_2_ atmosphere at 37 °C. Cells were cultured to confluency (about 80% density), detached using trypsin/versene (1/9 v/v), and then seeded in 24-well plates. Toroid-shaped gels were prepared and sterilized using 70% ethanol for 2 min prior to rehydration with DMEM for 2 h. DMEM was then removed, and the cells were seeded in 24-well plates at a density of 5 × 10^3^ cells per well. Control wells did not contain any gel. Cell cultures were then incubated in a humidified 5% CO_2_ atmosphere at 37 °C, and the medium was changed every 2 days. Live-dead assays were conducted on samples with or without the gels. After 7 days of culture, samples were incubated in a humidified 5% CO_2_ atmosphere at 37 °C in the presence of 100 μL Hoechst 33342 dye (10 μM), ethidium homodimer-I (4 μM), and calcein-AM (2 μM) in phosphate-buffered saline (PBS). After staining, images were recorded using a Leica DFC9000 GT sCMOS fluorescence microscope.

Alamar Blue assays were performed using L929 fibroblast cells. The gels were placed in 96-well tissue culture plates, and the cells were seeded on each gel at a density of 5 × 10^3^ cells/well. The plate was then placed in a CO_2_ incubator for 1, 3, and 7 days. Metabolic activity was assessed with Alamar Blue as specified by the manufacturer. Briefly, cells in each well were incubated with 15 μL Alamar Blue solution (10% v/v of serum-free media) for 3 h. A 150 μL solution from each well was then transferred to another 96-well plate for ELISA analysis at a wavelength of 570 nm.

## Results and Discussion

### Establishing Covalent Conductive Coacervate Gels

The NG used in this study had a number-average diameter, *D*_TEM_, of 44 ± 7 nm (Figure S1A) and contained 64 wt % MAA from potentiometric titration data (Figure S1B). The NGs had a p*K*_a_ of 6.4 and swelled when the pH was increased (Figure S1C). The NG particles are anionic as deduced from zeta potential data (Figure S1D). SAXS data for a concentrated NG dispersion (20 wt %) indicated a center-to-center distance of 52 nm (Figure S1E). The coacervate PEI–PEG gel was prepared as a control (Scheme S1). SAXS patterns for the noncovalently cross-linked PEI-NG indicate a mean center-to-center NG interparticle separation distance of 52 to 63 nm (Figure S2A). The PEI-NG had a compressive strength of more than 5.6 × 10^3^ kPa (Figure S2B). Young’s modulus is 10.5 kPa, while the tensile breaking strain is 1045% (Figure S2C). From dynamic tensile data (Figures S2D), the resilience (% work of deformation recovered upon strain removal) was 40–45% (Figure S2E). Additional discussion for the NGs and PEI-NG is provided in the Supporting Information.

**Figure 1:**
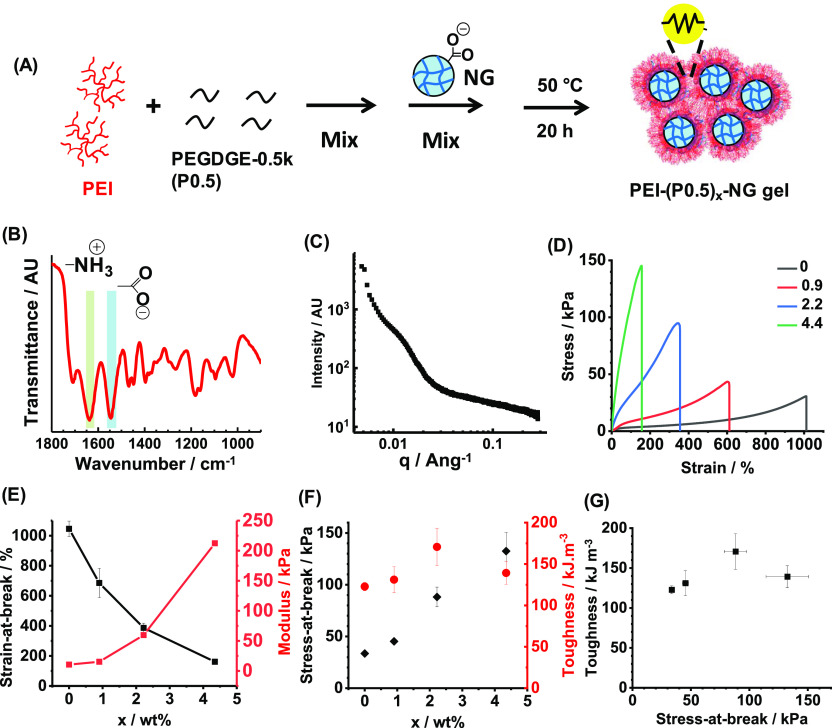
(A) Depiction of the preparation of PEI-(P0.5)_*x*_-NG. (B) FT-IR spectrum recorded for the PEI-(P0.5)_4.4_-NG after cross-linking. Vibrational bands assigned to ionic groups are shown. (C) SAXS pattern recorded for PEI-(P0.5)_4.4_-NG. (D) Tensile stress–strain data obtained for PEI-(P0.5)_*x*_-NGs. The legend shows the *x* values. (E) Strain-at-break and modulus. (F) Stress-at-break and toughness data for the gels. (G) Data from (F) replotted to show the relationship between toughness and gel strength.

To introduce covalent linking, we first incorporated PEGDGE-0.5k (P0.5) into the PEI-NGs to prepare PEI-(P0.5)_*x*_-NGs, where *x* denotes the P0.5 concentration used (in wt %)—see Figure 1A. P0.5 is a low-molecular-weight PEGDGE with a low epoxy equivalent weight (EEW). The EEW is the mass of the material containing 1 mol of epoxide, which is ∼250 g for P0.5. To probe the interactions present, an FT-IR spectrum was recorded for the PEI-(P0.5)_4.4_-NG (Figure 1B). The bands assigned to −NH_3_^+^ and −COO^–^ groups are indicative of ionic cross-linking between the PEI and NGs, which is a signature of the coacervate state depicted in Scheme 1.^43^ The shoulder at 915 cm^–1^ that was present in the spectrum for the P0.5 cross-linker due to epoxy bending was absent in the gel, suggesting that these groups had reacted.^47^ (Full FT-IR spectra for the gel and the components appear in Figure S3.) Scheme S2 depicts the epoxy-amine cross-linking that is proposed to occur. (This aspect is revisited below.) SAXS was used to probe the structure of the PEI-(P0.5)_4.4_-NG (Figure 1C). The scattering pattern is similar to that recorded for PEI-NG (Figure S2A), suggesting similar arrangements for the NG and PEI components in this covalently cross-linked coacervate gel. The shoulder observed at *q* = 0.0115 Å^–1^ indicates a mean center-to-center separation distance of 55 nm. This is sufficient to accommodate the branched PEI chains between neighboring NG particles.

The inclusion of P0.5 has a dramatic effect on the tensile stress–strain properties exhibited by the corresponding PEI-(P0.5)_*x*_-NGs (Figure 1D). The gels become stiffer and less stretchable as *x* is increased from 0 to 4.4. The modulus increases by a factor of 20 (from 10.5 to 212 kPa), and the strain-at-break is reduced from 1045 to 161% (see Figure 1E). The stress-at-break increased with increasing *x*; while the toughness reached a maximum for *x* = 2.0% (Figure 1F). This behavior is consistent with covalent cross-linking owing to the reaction of P0.5 with the primary amine groups on the PEI chains (Scheme S2). However, the toughness did not increase significantly with the stress-at-break (i.e., gel strength) for this series of gels shown by Figure 1G.

**Figure 2:**
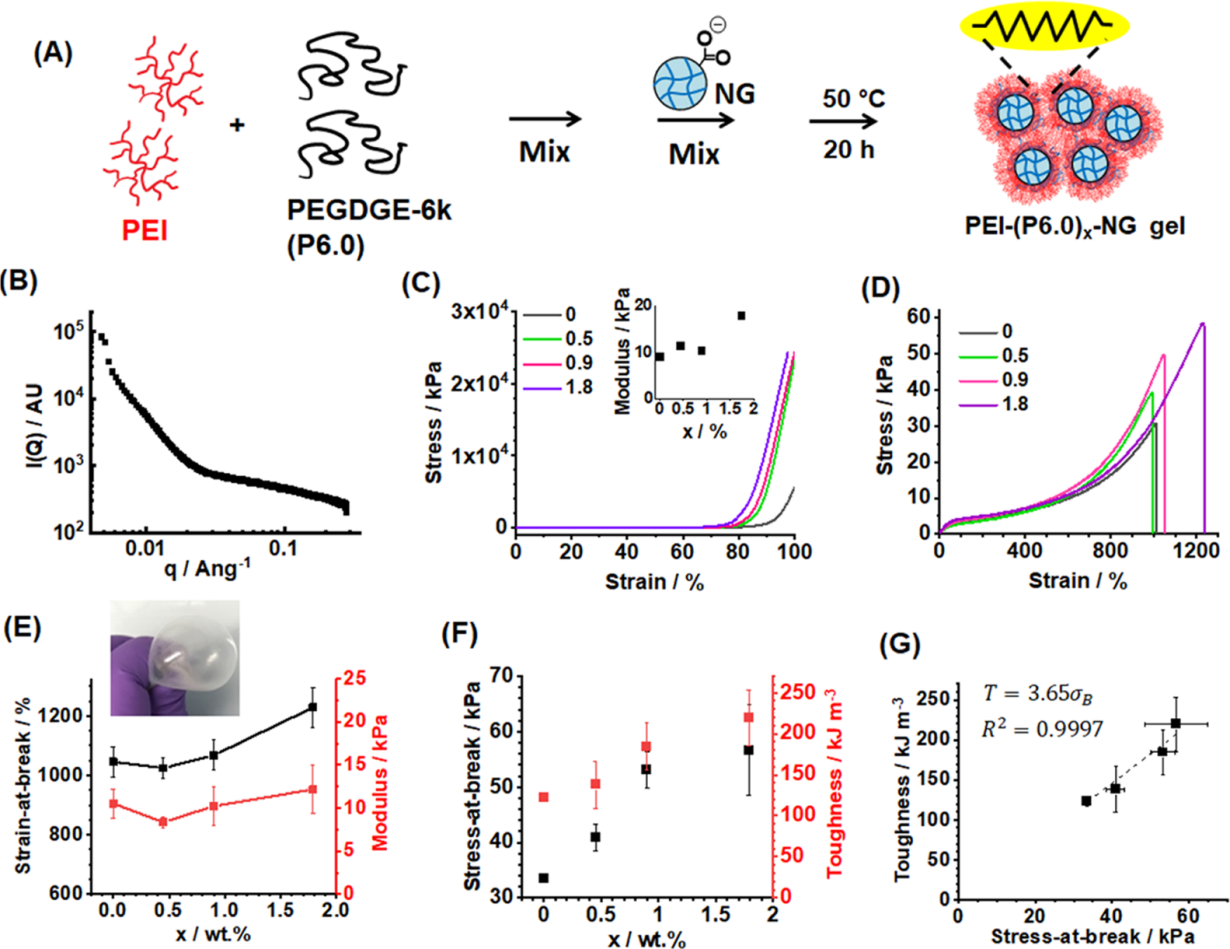
(A) Depiction of the preparation of PEI-(P6.0)_*x*_-NGs. (B) SAXS pattern recorded for PEI-(P6.0)_0.9_-NG. (C) Compression stress–strain data recorded for the gels. The inset shows the compressive moduli for the gels. (D) Tensile stress–strain data obtained for a series of PEI-(P6.0)_*x*_-NGs. The legend shows the *x* values in wt %. (E) Strain-at-break and modulus. The inset shows a balloon prepared from the PEI-(P6.0)_0.9_-NG. (F) Stress-at-break data and toughness data for the gels. (G) Data from (F) replotted to show relationship between gel toughness and strength.

We therefore investigated the effect of increasing the PEGDGE chain length by replacing P0.5 with P6.0 (see Figure 2A). The latter has an EEW of ∼3000 g, which is a factor of 12 larger than that for P0.5. SAXS data were obtained for the PEI-(P6.0)_0.9_-NG (Figure 2B). Compared to the SAXS pattern recorded for a PEI-(P0.5)_4.4_-NG (Figure 1B), there is less structural order for the PEI-(P6.0)_0.9_-NG. The weak shoulder at *q* ∼ 0.010 Å^–1^ corresponds to a mean inter-NG distance of 63 nm. The less-prominent nature of this feature is attributed to the relatively large P6.0 chains, which would be less readily able to be packed evenly within the gel. The PEI-(P6.0)_*x*_-NGs displayed excellent mechanical performance when subjected to compression (Figure 2C). A stress of 2.4 × 10^4^ kPa at 98% strain was achieved for the PEI-(P6.0)_1.8_-NG without any signs of fracture. This inability to fracture prevented determination of the compression stress-at-break and strain-at-break values. The compression moduli are shown in the inset of Figure 2C and generally increase with increasing *x*.

Tensile stress–strain data measured for PEI-(P6.0)_*x*_-NGs showed that using P6.0 produced increasingly stretchable gels (Figure 2D). Interestingly, the strain-at-break increased from 1045 to 1230% as *x* was increased from 0 to 1.8% (Figure 2E). This is in striking contrast to the trend observed for the PEI-(P0.5)_*x*_-NGs (Figure 1E). Hence, introduction of a P6.0-based network helps to prevent crack propagation. Indeed, this enabled a balloon of PEI-(P6.0)_0.9_-NG to be inflated (inset of Figure 2E). We attribute this to the relatively long PEGDGE chains. It is proposed that there is a switch from intra- and inter-PEI cross-linking for PEI-(P0.5)_*x*_-NGs to predominantly inter-PEI cross-linking for PEI-(P6.0)_*x*_-NGs due to the large size and greater exclusion from the PEI interior of P6.0 compared to that of P0.5. It follows the fact that the ability of the PEI chains to stretch should be reduced as *x* increases for the PEI-(P0.5)_*x*_-NGs. In contrast, the PEI-(P6.0)_*x*_-NGs would not be directly affected. Furthermore, the inter-PEI linkages based on P6.0 should be more stretchable than those for P0.5 due to the longer P6.0 chain length. This mechanism implies that a significant structural difference should exist between the PEI-(P6.0)_*x*_-NG and PEI-(P0.5)_*x*_-NGs. This interpretation is supported by the respective SAXS profiles (Figures 1C and 2B) as discussed above. One reviewer of this article suggested that a greater number of entanglements for the PEI-(P6.0)_*x*_-NGs may contribute to the differences in mechanical properties; we agree that such an explanation is plausible. Furthermore, there should be fewer chemical cross-links within the PEI-(P6.0)_*x*_-NGs compared to the PEI-(P0.5)_*x*_-NGs due to the much higher-molecular-weight PEGDGE used in the former.

The Young modulus was not significantly affected by *x* for the PEI-(P6.0)_*x*_-NGs (Figure 2E). Interestingly, both the stress-at-break and the toughness increase with increasing *x* (Figure 2F). Indeed, Figure 2G reveals that the toughness increases linearly with increasing stress-at-break for these gels. This result shows that increasing the PEGDGE length provides these gels with the paradoxical (and rare) property that their toughness increases with increasing strength.

Cyclic tensile stress–strain properties of the PEI-(P6.0)_0.9_-NG were investigated (see Figure S4A). The data (Figure S4B) revealed that the resilience increased and residual strain decreased compared to the parent PEI-NGs (Figure S2E). It is proposed that this P6.0-based covalent network contains relatively long strands (depicted in Figure 2A), which are responsible for the highly stretchable nature of the PEI-(P6.0)_*x*_-NGs and also their improved resilience. We investigated PEI-to-P6.0 covalent cross-linking using a range of NG-free solutions using vial inversion (Figure S5A) and FT-IR spectroscopy (Figure S6). The vial inversion data show strong evidence for covalent cross-linking between PEI and P6.0 and are discussed in detail in the Supporting Information. The coacervate state occurs when the anionic NGs locally concentrate PEI (and P6.0). We propose that covalent cross-linking via the reaction of primary amines with the PEGDGE epoxide groups occurs in the PEI domains within the covalent complex coacervate PEI-(P0.5/P6.0)_*x*_-NGs.

Having established that P6.0 gave improved mechanical properties that defied the toughness-strength paradox, we then sought to include an electrically conductive additive into the PEI-(P6.0)_0.9_-NG. Although Gnps can be dispersed in polar solvents,^42^ they are only poorly dispersed in water. Pleasingly, we discovered that adding Gnps to the PEI/P6.0 mixture prior to the addition of the NGs (Scheme 1) greatly improved their aqueous dispersibility and colloid stability (see Figure S7). SEM images obtained for the Gnps indicated particles in the size range of ∼0.5–2 μm (Figure S8). Furthermore, SEM confirmed the presence of Gnps of ∼2 μm diameter within a freeze-dried PEI-(P6.0)_0.9_-NG/Gnp_3.1_ gel, as shown in Figure S9. The FT-IR spectrum recorded for PEI-(P6.0)_0.9_-NG/Gnp_3.1_ (Figure S10) is similar to that obtained for PEI-(P0.5)_4.4_-NG (Figure 1B), with bands indicative of ionic bonding between RCOO^–^ and RNH_3_^+^ present at 1552 and 1637 cm^–1^.

**Figure 3:**
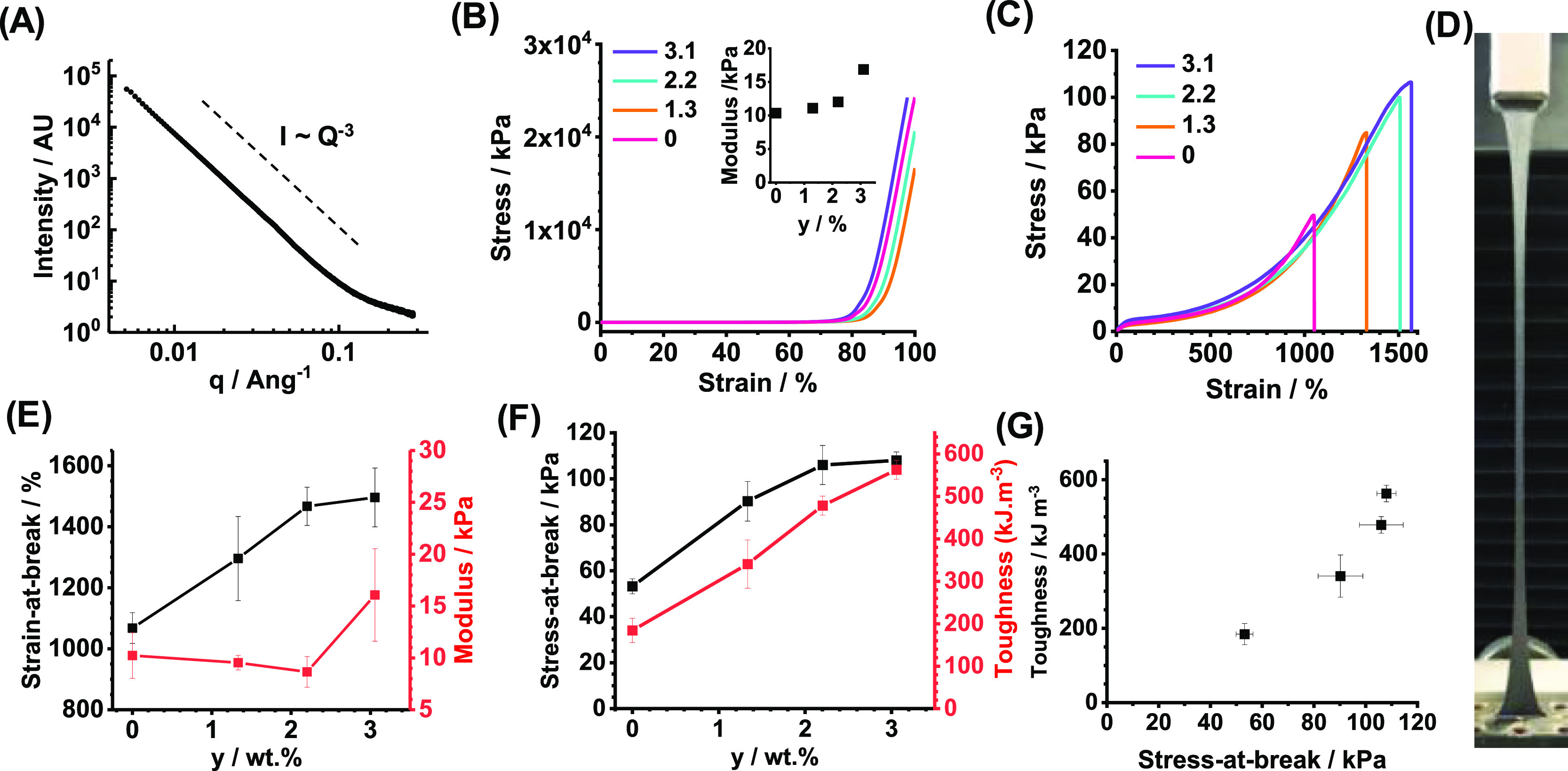
(A) SAXS pattern for PEI-(P6.0)_0.9_-NG/Gnp_3.1_. (B) Compression stress–strain data recorded for PEI-(P6.0)_0.9_-NG/Gnp_*y*_ gels. (C) Tensile stress–strain data obtained for PEI-(P6.0)_0.9_-NG/Gnp_*y*_ gels. The legend shows the *y* values in wt %. (D) Photograph of a stretched PEI-(P6.0)_0.9_-NG/Gnp_3.1_ gel. (E) Breaking strain and modulus. (F) Stress-at-break and toughness for the gels. (G) Data from (F) plotted to show the relationship between toughness and strength for these conductive gels.

We employed SAXS to probe the structure of a PEI-(P6.0)_0.9_-NG/Gnp_3.1_ gel, see Figure 3A. The scattering pattern is profoundly different to those recorded for the gels not containing Gnps (compared to Figures 1C and 2B). The scattering for this gel is dominated by a Porod component with a scattering exponent of −3.0, that is, *I*(*q*) ∼ *q*^–3^. This corresponds to scattering from objects with a very rough surface^48^ and is due to the Gnps. These data show that the Gnp surface had a major effect on the structure of these gels. The Gnps did not adversely affect the compression behavior for the gels, and the failure strains for the PEI-(P6.0)_0.9_-NG/Gnp_*y*_ gels exceeded the upper limit for our instrument (Figure 3B). The compressive strain-at-break values are more than 97.5%. The compression moduli (inset of Figure 3B) increased with the increasing Gnp content (*y*).

Tensile stress–strain data were measured for the PEI-(P6.0)_0.9_-NG/Gnp_*y*_ gels (Figure 3C). The gels are highly stretchable, as can be seen in Figure 3D. Remarkably, the strain-at-break increases with increasing *y* (Figure 3E). Indeed, the strain-at-break increased from 1065 to 1500% as *y* was increased from 0 to 3.1%. The gel modulus remained almost constant until *y* = 3.1%, whereby an increase from 10.2 to 16.1 kPa was observed (Figure 3E). The stress-at-break (Figure 3F) initially increased with *y* and then reached a plateau for *y* = 3.1%. The gel toughness increases with increasing *y* (Figure 3F) owing to the combined increases in strain-at-break (Figure 3E) and stress-at-break. The toughness is plotted against stress-at-break in Figure 3G, and these data show a supra-linear relationship. Hence, inclusion of the Gnps further amplified the rare phenomenon, whereby the toughness increased for these conductive gels as their strength increased. Dynamic tensile measurements for PEI-(P6.0)_0.9_-NG/Gnp_3.1_ were conducted (Figure S11). The data show a higher residual strain and lower resilience than that for the Gnp-free parent gels (Figure S4). This is attributed to the Gnps which dissipate energy as they move past one another under strain. The ability of the Gnps to increase energy dissipation likely contributes to the improved toughness of these gels.

The softness and deformability of the NG are also likely to be important in controlling the mechanical properties of these gels. While investigating the effect of cross-linker concentration on the mechanical properties of the current gels is beyond the scope of this article, some comments are appropriate. The NGs selected for this work can swell strongly (Figure S1C) and have provided covalently interlinked microgel gels with excellent ductility.^49^ Increasing the intra-NG cross-link concentration would likely stiffen the NG particles. Such effects are well known for microgels.^50^ Stiffer NGs are likely to increase the gel modulus and decrease the strain-at-break.

### Investigating PEI-(P6.0)_0.9_-NG/Gnp_3.1_ Gels for Strain Sensing

The PEI-(P6.0)_0.9_-NG/Gnp_3.1_ gel had the highest toughness and strength (Figure 3F) and was therefore selected to be tested as a potential sensor for electronic skin. To act as a sensor for electronic skin applications, the gel should be adhesive to enable the formation of a conformal coating. We measured the adhesive strength of this gel to a poly(methyl methacrylate) plate and compared the data to those measured for PEI-NG and PEI-(P6.0)_0.9_-NGs. The adhesive strength for the PEI-(P6.0)_0.9_-NG/Gnp_3.1_ was 16.1 kPa. This is higher than those for PEI-NG (14.7 kPa) and PEI-(P6.0)_0.9_-NG (13.6 kPa) (see Figure S12). In recent work, it was shown that PEI-NGs adhered to a range of substrates including porcine skin.^43^

**Figure 4:**
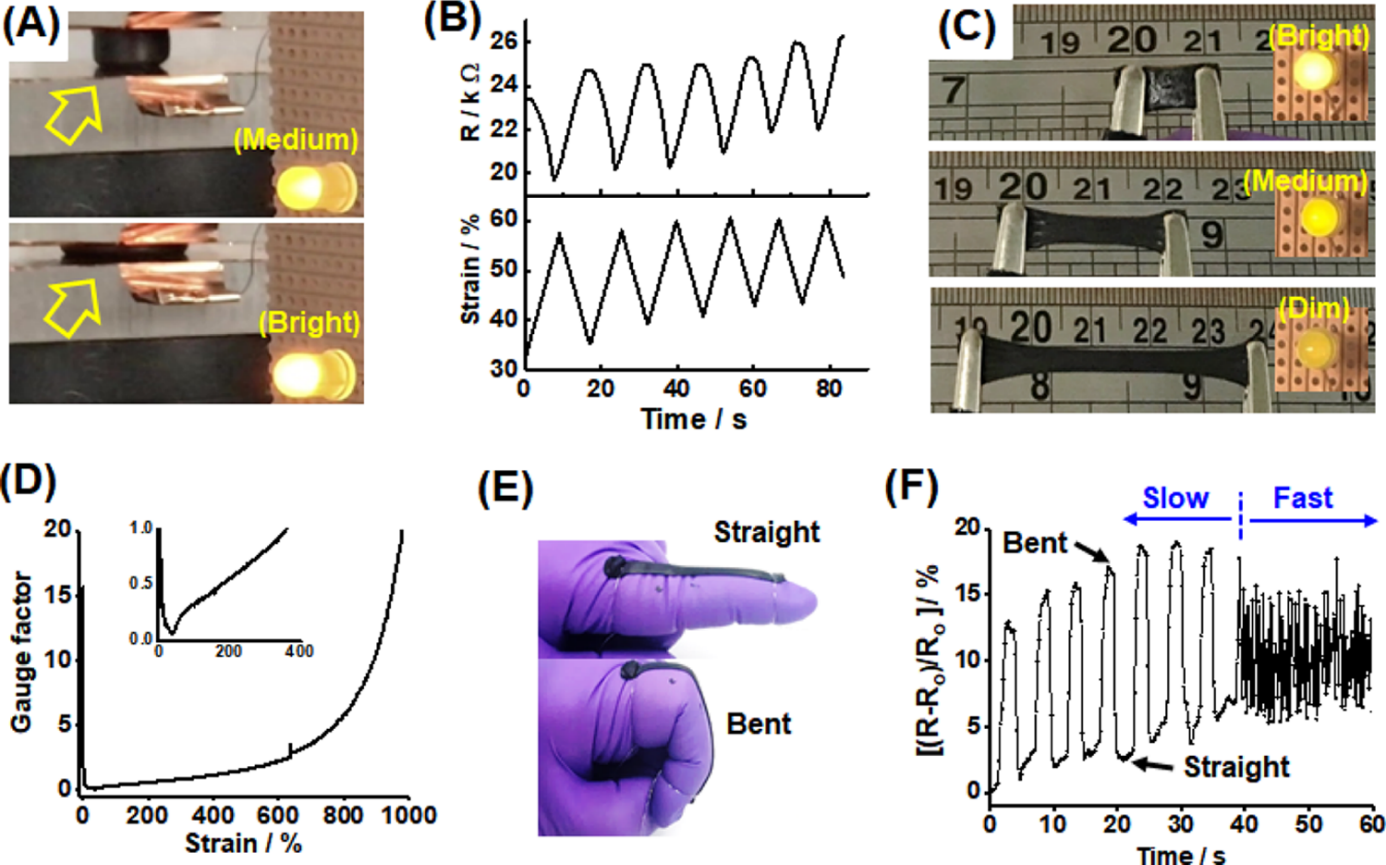
Investigating PEI-(P6.0i)_0.9_-NG/Gnp_3.1_ gel as a strain sensor. (A) Compression experiment showing that this cylindrical gel (see the yellow arrow) becomes more electrically conductive (brighter illumination) when subjected to a compressive strain. (B) Variation in gel resistance over six compression cycles. (C) Tensile strain-sensing experiment using an LED. (D) Gauge factor vs tensile strain. The inset shows an expanded view. (E) Self-adhesive gel attached to a gloved finger and secured with two wires. (F) Movement sensing of a finger during slow and fast bending.

The PEI-(P6.0)_0.9_-NG/Gnp_3.1_ gel was investigated for potential use as a strain sensor. Figure 4A shows its pressure-dependent change in electrical resistance in terms of the variation in light intensity from a light-emitting diode (LED). A cylindrical gel was placed between two copper plates connected to an electrical circuit containing an LED, and a voltage was applied. The LED brightness increased as the gel was compressed (see Movie S1 in the Supporting Information). The resistance and compressive strain were also measured (Figure 4B), with minimum resistance being observed at maximum compressive strain. (A possible explanation for the drift in these data is partial dehydration of the gels.) This is consistent with Figure 4A and indicates a greater number of percolating pathways being formed within the compressed gel. The change in resistance (∼4 kΩ) observed for a ∼20% increase in strain suggests that this device has potential for development as a pressure sensor.

The response of a stretched gel was also investigated using an LED (see Figure 4C and Movie S2 in the Supporting Information). The LED light intensity decreases as the gel is stretched and increases immediately after the removal of the tensile force. This demonstrates the ability of the hydrogel to sense tensile strain. The gauge factor ([Δ*R*/R]/strain) during tension was determined (Figure 4D). After an initial reduction, there is a linear region from 70 to 380% strain, after which the gauge factor increases exponentially. This linear region bodes well for potential applications, while the exponential region provides enhanced sensitivity for large strains. The latter response is due to a reduction in the number of percolating electrical pathways within the gel. The electrical circuit was broken by cutting the gel in half. LED illumination was observed on bringing the two halves of the gels back into contact, which indicates that these gels are electrically self-healable (Figure S13).

The ability to sense human motion is highly desirable for flexible wearable electronic devices.^51^ Noting that parent PEI-NGs are adhesive to porcine skin,^43^ an adhesive gel was placed on a rubber glove to create a conformal contact, which is ideal for such sensing applications.^52^ The real-time change in resistance that results from the movement of an index finger indicates that these gels can detect large-scale human motion corresponding to ∼500% strain (see Figure 4E,F). A relative change in the resistance of ∼15% is observed for slow finger bending, which compares well with other conductive stretchable hydrogels.^12^ The drift evident during slow bending may possibly be due to partial dehydration which could, in principle, be removed by encapsulating the gel. The data show that the gel sensor was also able to monitor fast finger bending and that the extent of bending during those cycles was less than that for the slow bending.

The drift effects noted above were evident within 90 s as a result of the rapid strain cycling exerted on these conducting hydrogels (Figure 4B,F). Drying is a well-known problem for hydrogels in open environments^53^ and can affect hydrogel sensors if not encapsulated. Sun et al. successfully encapsulated their carbon nanotube-based sensor in commercial VHB tape^21^ and achieved sensing with low or negligible drift over extended periods. Such an approach could also be used for our PEI-(P6.0)_0.9_-NG/Gnp_3.1_ gels.

Having established the ability of the PEI-(P6.0)_0.9_-NG/Gnp_3.1_ gel to report compressive and tensile strain, we investigated the swelling behavior after it was immersed in PBS buffer (pH 7.4). The volume swelling ratio (*Q*_*v*_) for this gel as well as PEI-(P6.0)_0.9_-NG and PEI-NG was measured before immersion (pH 8.6–9.8) and after reaching swelling equilibrium in PBS (see Figure S14). The *Q*_*v*_ value for noncovalently cross-linked PEI-NG decreased from 2.6 to 1.4 as the pH decreased in line with the earlier report.^43^ In contrast, the *Q*_*v*_ values for both PEI-(P6.0)_0.9_-NG and PEI-(P6.0)_0.9_-NG/Gnp_3.1_ increased from 2.7 to 4.4 and 2.3 to 2.7, respectively, as the pH decreased to 7.4. The large-scale structural rearrangements that occurred for PEI-NG upon changing the pH^43^ were not possible for our covalently cross-linked gels. For both PEI-(P6.0)_0.9_-NG and PEI-(P6.0)_0.9_-NG/Gnp_3.1_, the *Q*_*v*_ values increased due to water ingress in order to decrease the osmotic pressure difference between the polyelectrolyte gel interior and the exterior solution. The relative *Q*_*v*_ value increase for PEI-(P6.0)_0.9_-NG/Gnp_3.1_ is less than that for PEI-(P6.0)_0.9_-NG due to the Gnps providing noncovalent cross-linking in the former gel. Evidence for this assertion can be found in the higher modulus values for PEI-(P6.0)_0.9_-NG/Gnp_3.1_ compared to those for PEI-(P6.0)_0.9_-NG (see Figure 3E).

**Figure 5:**
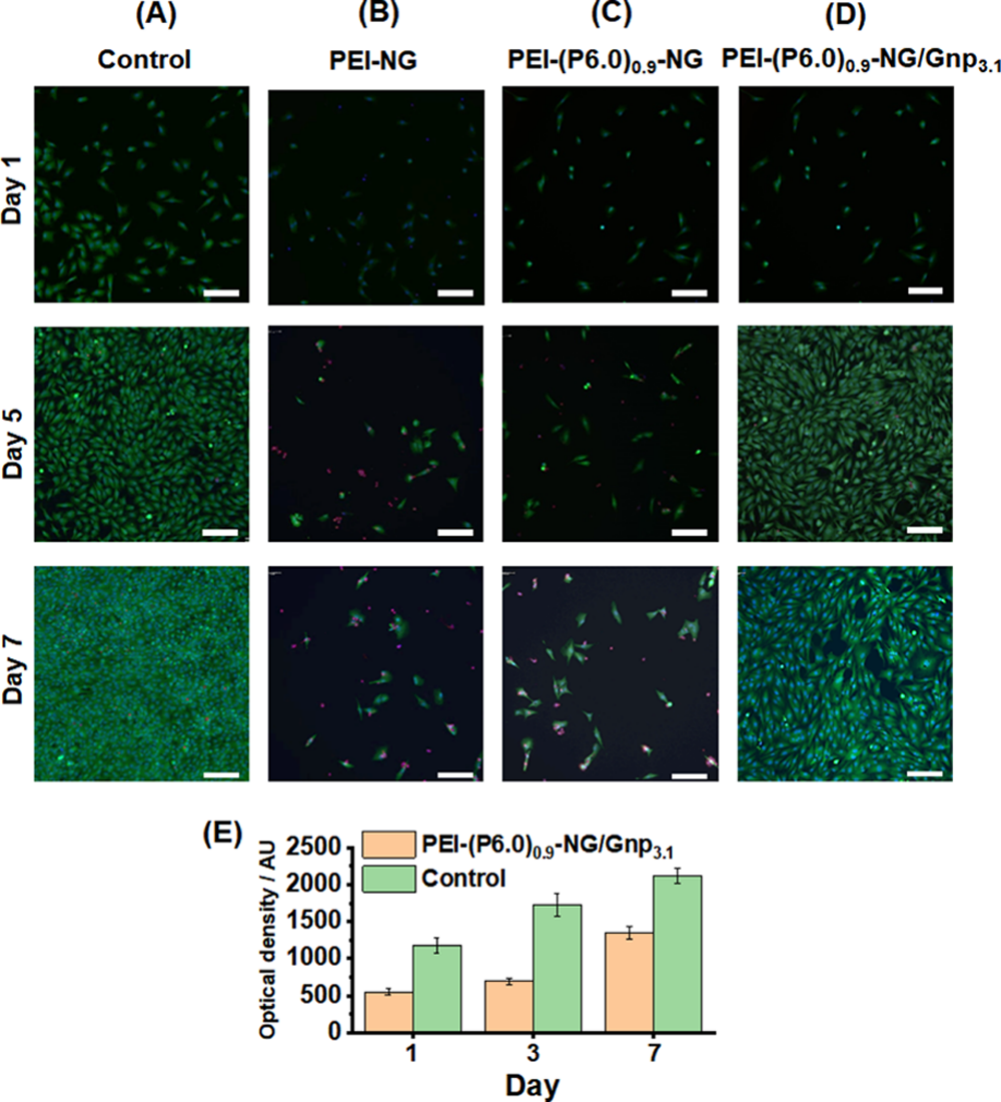
(A–D) Live-dead assay images for human fibroblast HCA2 cells in the presence of various gels recorded after 1, 5, and 7 days (scale bar = 100 μm in each case). Green and red cells are live and dead, respectively. (E) Alamar Blue assay data obtained for fibroblast L929 cells when using PEI-(P6.0)_0.9_-NG/Gnp_3.1_ gel and a control.

Cell viabilities for both HCA2 and L929 fibroblast cells were evaluated in the presence of all of the gels and a gel-free control (see Figure 5A–D). Accordingly, cells were stained for a live/dead assay. Some dead cells are evident for PEI-NG (Figure 5B) and PEI-(P6)_0.9_-NG (Figure 5C), which is likely due to free PEI. Importantly, there are very few dead cells for the conductive PEI-(P6.0)_0.9_-NG/Gnp_3.1_ (Figure 5D) gel, which indicates that there was little, if any, free PEI for that system and that the HCA2 cells remained viable. Figure 5E shows the optical density, which is directly related to the cell viability of L929 cells, for the PEI-(P6.0)_0.9_-NG/Gnp_3.1_ gel compared to a polystyrene control. Although the optical density was lower than that of the control, good cytocompatibility was demonstrated and the cell density increased strongly between day 3 and day 7. These results are encouraging for biomaterial applications, including sensing, of our new conductive gels.

## Conclusions

We report a facile new protocol for the preparation of highly stretchable electrically conductive gels that defy the toughness-strength paradox. The molecular weight of the PEGDGE cross-linker plays an important role in determining the mechanical properties of the resulting gels. Thus, using a PEGDGE of 6000 g mol^–1^ rather than 500 g mol^–1^ leads to a softer, more stretchable gel with toughness values that increase with stress-at-break (Figure 2G). Remarkably, addition of Gnps not only confers electrical conductivity but also further increases gel toughness and strength, enabling gels to be stretched to up to 1500% of their original dimensions. Our data demonstrate that covalent linking of P6.0 with PEI plays a crucial role in improving the mechanical properties and swelling behavior. The excellent physical properties of these electrically conductive hybrid gels are exploited to demonstrate pressure and tensile strain-dependent sensing that can respond rapidly to human motion. Moreover, gel preparation is efficient, cost-effective, and amenable to industrial scale-up. The PEI-(P6.0)_0.9_-NG/Gnp_3.1_ gel is shown to have good cytocompatibility with fibroblast cells. To the best of our knowledge, this is the first reported use of polycarboxylated Gnps for the synthesis of stretchable and conductive hydrogels.

## Supporting Information Available

PEI-(P6.0)_0.9_/NG/Gnp_3.1_ gel being compressed or stretched and the effects of LED illumination, discussion and characterization data for the NGs and also PEI-NGs, synthesis of PEI-NG and the covalent cross-linking reaction, FT-IR spectra for all of the systems studied, cyclic tensile and resilience data, vial inversion images and FT-IR spectra for the PEI/P6.0 control systems and gels, photographs of PEI/Gnp dispersions, SEM of PEI/Gnp mixtures and freeze-dried PEI-(P6.0)_0.9_/NG/Gnp_3.1_ gel, adhesion data for the gels, electronic self-healing experiment photographs, swelling data for the gels, and summarized SAXS data (PDF)
Increase of LED brightness as the gel was compressed (MOV)
Response of the stretched gel using an LED (MOV)

## Supplementary Material

bm1c01660_si_001.pdf

bm1c01660_si_002.mov

bm1c01660_si_003.mov
