# Supplementary material for: Highly Stretchable Conductive Covalent Coacervate Gels for Electronic Skin
Source: Biomacromolecules. 2022 Feb 21;23(3):1423–32. doi: 10.1021/acs.biomac.1c01660 (PMC9098112; doi:10.1021/acs.biomac.1c01660)
Supplement: Supplementary file 1 — bm1c01660_si_001.pdf [file bm1c01660_si_001.pdf]

SUPPORTING INFORMATION for

Highly-stretchable conductive covalent coacervate gels for electronic skin

Nam T. Nguyen[a,*], James Jennings[b], Amir H. Milani[a], Chiara D. S. Martino[c], Linh T. B. Nguyen[d], Shanglin Wu [a], Muhamad Z. Mokhtar[a], Jennifer M. Saunders[a], Julien E. Gautrot [c], Steven P. Armes[b] and Brian R. Saunders.[a,*]

[a] *Department of Materials, University of Manchester, MSS Tower, Manchester, M13 9PL, U.K*

[b] *Department of Chemistry, University of Sheffield, Sheffield, S3 7HF, United Kingdom.*

[c] *School of Engineering and Materials Science, Queen Mary University of London, London, E1 4NS, United Kingdom*

[d] *Eastman Dental Institute, University College London, London, WC1X 8LD, United Kingdom*

Legend for Supplementary Movies

Movie S1

Compression sensing

Video showing the response of an LED connected in series with a battery and a cylindrical PEI-(P6.0)$_{0.9}$-/NG/Gnp$_{3.1}$ gel cylinder as this is compressed and released.

Movie S2

Tension sensing

Video showing the response of an LED connected in series with a battery and a PEI-(P6.0)$_{0.9}$-/NG/Gnp$_{3.1}$ gel film as this is stretched and released

Supplementary schemes

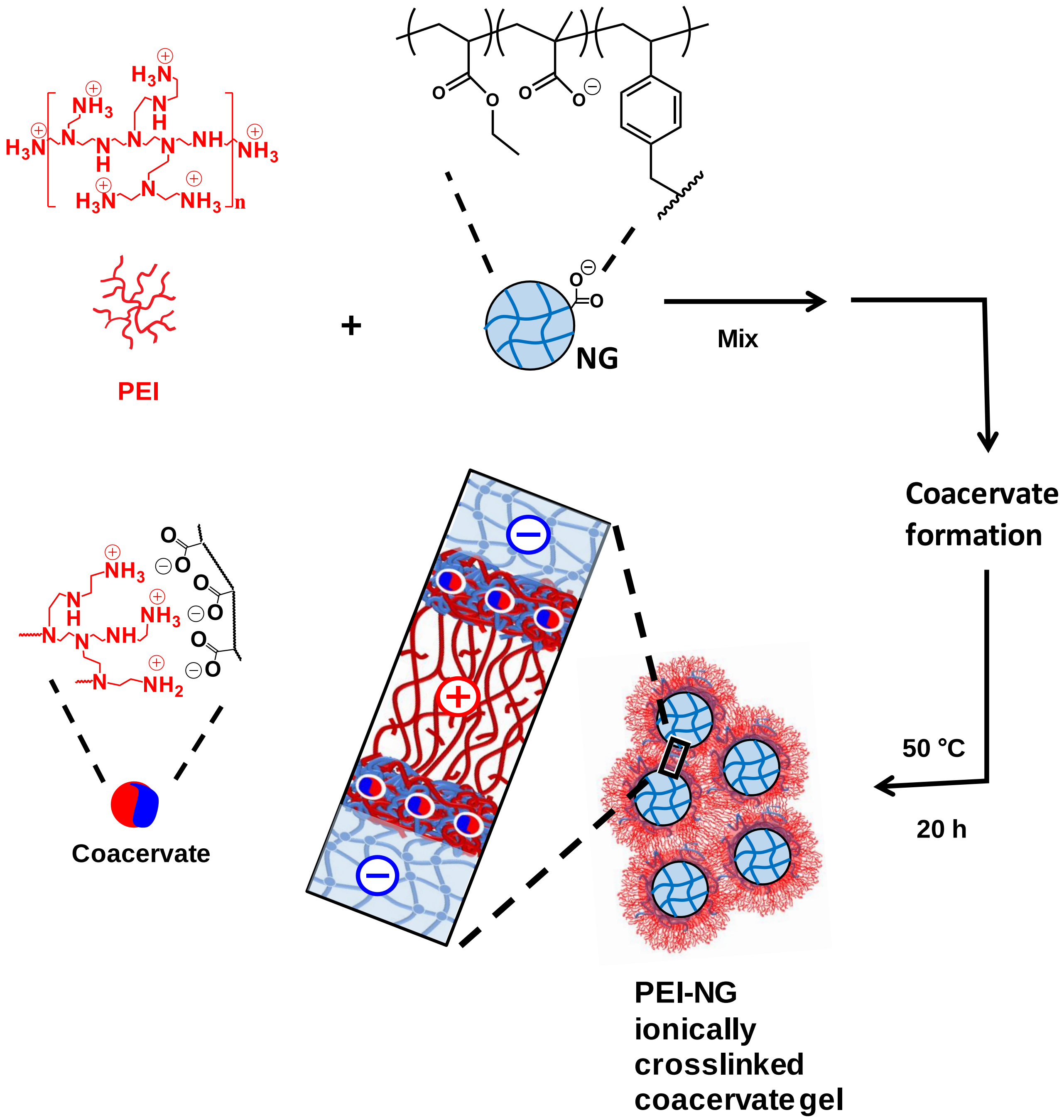

Scheme S1. Depiction of the assembly of the non-covalently crosslinked complex coacervate PEI-NG gel.

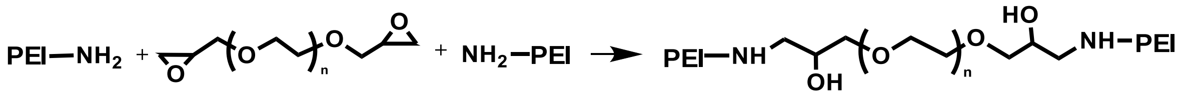

Scheme S2. Depiction of the reaction between PEI and PEGDGE.

Supplementary additional discussion and figures

Nanogel characterization

We first synthesized pH-responsive poly(EA-MAA-DVB) NG particles by aqueous emulsion polymerization.[1] TEM studies indicated a number-average diameter, D_{TEM}, of 44 ± 7 nm (Figure S1A). Acid titration data (Figure S1B) indicated that these NG particles contained 64 wt.% MAA and had a pK_a of 6.4. According to DLS studies, the intensity-average NG diameter increased from 69 nm to 271 nm when raising the solution pH from 5.8 to 9.8 (Figure S1C). The NG particles acquire anionic character owing to ionization of the MAA repeat units, with the zeta potential becoming increasingly negative as the solution pH exceeds the pK_a (Figure S1D). A SAXS pattern recorded for a concentrated aqueous dispersion (20 wt.%, Figure S1E) contains a structure peak at a scattering vector (q) of 0.012 $Å^{-1}$ with a corresponding centre-to-centre distance ($L = 2\pi/q$) of 52 nm. The latter value agrees well with the average inter-NG separation of 60 nm calculated from[2] $L = N_d^{1/3}$ using the number density of particles (N_d) and a diameter of 44 nm from TEM. All the SAXS data obtained in this study are summarized in Table S1.

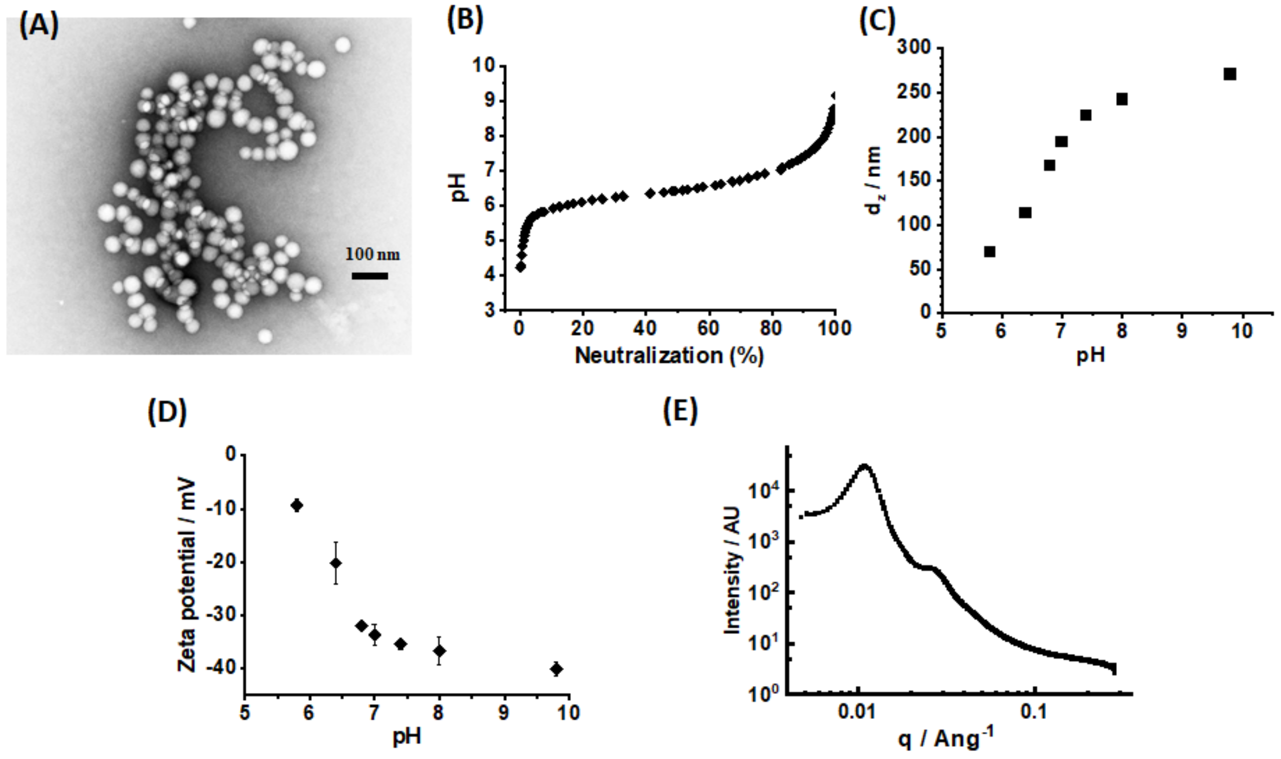

Figure S1. Characterization of poly(EA-co-MAA-co-DVB) NG. **(A)** Representative TEM image. **(B)** Potentiometric titration data. **(C)** Variation of the z-average diameter (d_z) with pH as measured by DLS. **(D)** Variation of zeta potential with pH. **(E)** SAXS data measured for a dispersion with a concentration of 20 wt.%. The pH of the dispersion was 3.9.

PEI-NG complex coacervate gel characterization

A PEI-NG gel was prepared as a control for this study. PEI-NG is a new type of coacervate gel[3] prepared by simply mixing PEI and NG and heating at 50 °C for 20 h (Scheme S1). These gels comprise NGs separated by branched PEI chains,[3] as depicted in Scheme S1. SAXS patterns for the gel contained a broad shoulder in the range q = 0.010 to 0.012 Å^{-1} corresponding to a mean centre-to-centre NG inter-particle separation distance (= $2\pi/q$) of 52 to 63 nm (Figure S2A). The compressive strength of the gel is more than 5.6 x 10^{3} kPa (Figure S2B) and its breaking strain (> 98%) under compression was beyond the upper limit value accessible with our instrument. This physical gel is highly stretchable with a breaking strain of 1045 % and a modulus of 10.5 kPa (Figure S2C). Dynamic tensile data (Figure S2D) showed a resilience (% work of deformation recovered upon strain removal) of 40 – 45 % (Figure S2E). The sacrificial ionic bonds between RNH_3^+, $R_2NH_2^+$, R_3NH^+ and $RCOO^-$ groups as well as the inherent deformability of PEI and NG account for the high breaking strain observed for the PEI-NG gel[3].

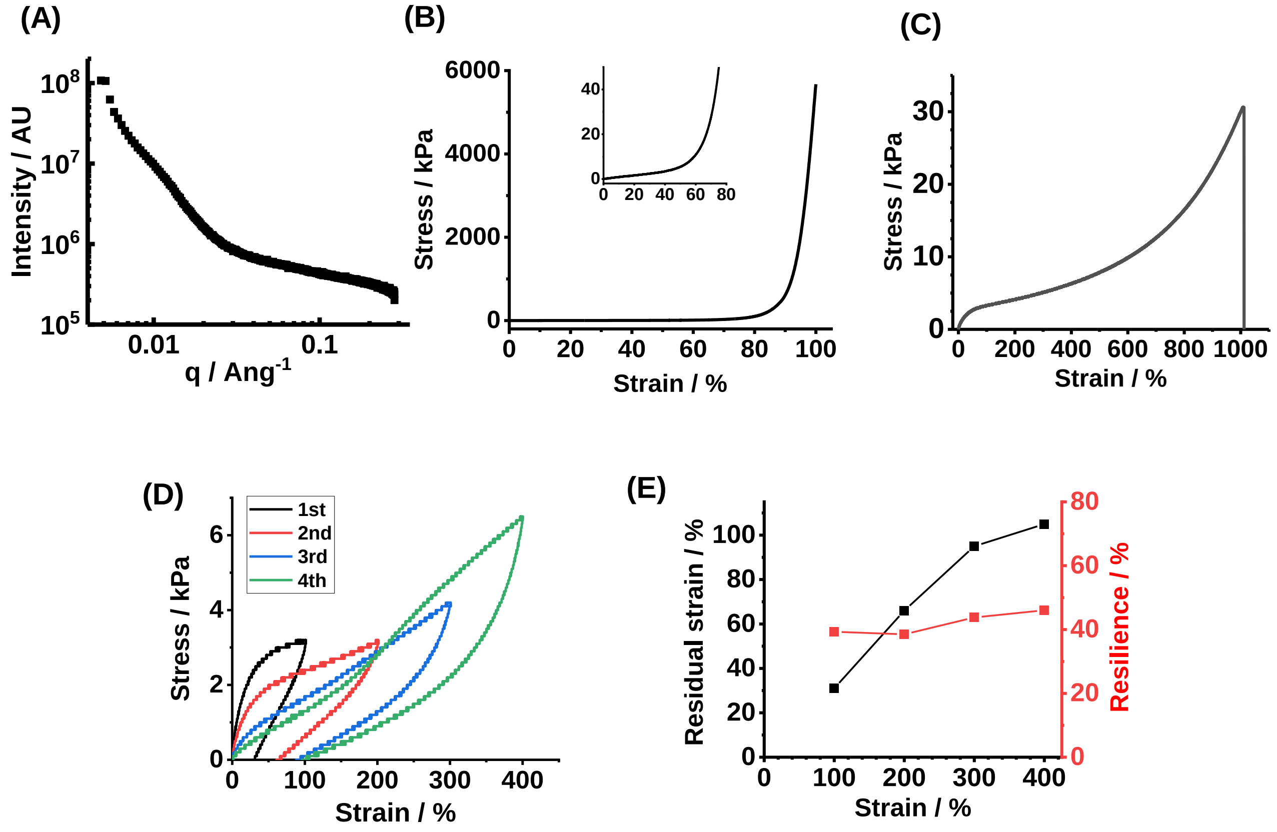

Figure S2 **(A)** SAXS data for PEI-NG gel. **(B)** Compression stress-strain data. The inset shows an expanded view of the low strain region. **(C)** Tensile stress-strain data. **(D)** Cyclic tensile data and **(E)** Residual strain and resilience from (D).

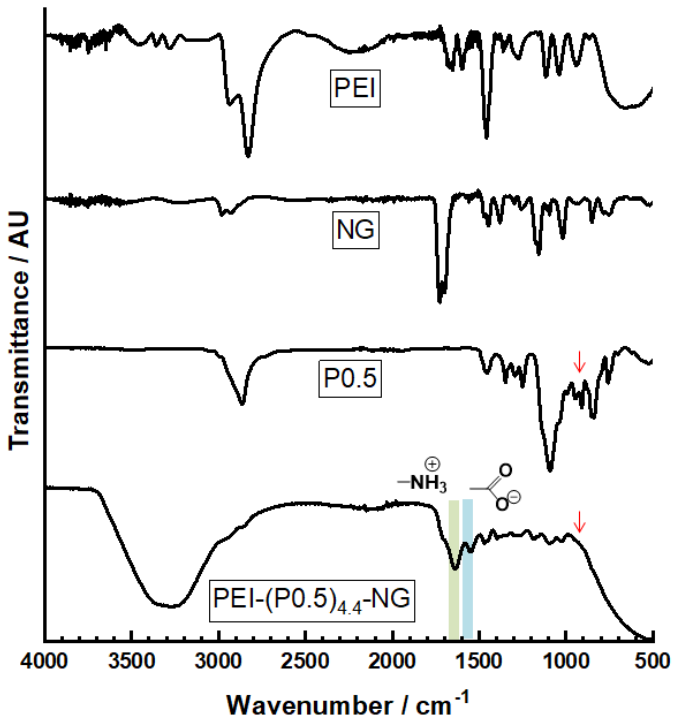

Figure S3. FTIR spectra PEI, NG and PEGDGE-0.5k (P0.5) and also PEI-$(P0.5)_{4.4}$-NG gel after mixing. Peaks due to the ionic groups are highlighted with green and blue shading. The position of the epoxide peak (915 cm^{-1}) is indicated with a red arrow.

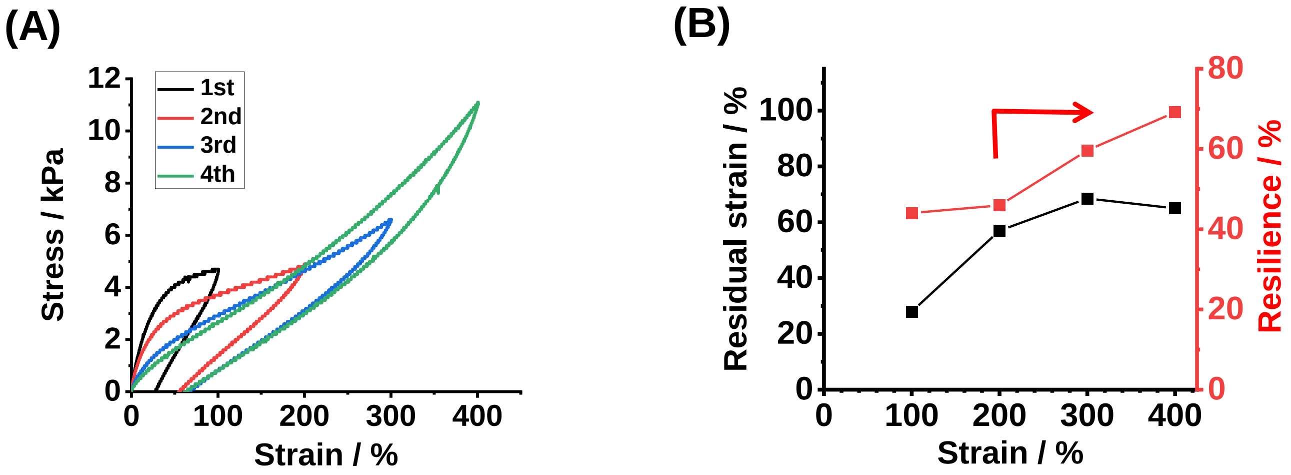

Figure S4. Cyclic tensile data **(A)** and residual strain and resilience **(B)** for PEI-(P6.0)$_{0.9}$-NG.

Evidence for chemical crosslinking of PEI by P6.0

In order to further investigate the mechanism for crosslinking a number of PEI/P6.0 solutions without NGs were prepared and heated at 50 °C for ~ 20 h and then examined using vial inversion for evidence of gelation (see Figure S5A). Interestingly, the PEI/P6.0 solution did not form a gel at the concentration used to prepare the PEI-$(P6.0)_{0.9}$-NG gel (see Vial E). An image for the latter gel is shown in Figure S5B for comparison. Importantly, when the PEI + P6.0 concentration was increased to ~ 50 wt.% at a similar epoxide-to-primary amine (1°) group ratio a gel formed provided this ratio was greater than or equal to 0.87 (Vial D). (It is assumed that $1/3^{rd}$ of the amine groups in PEI are primary amines[4].) These data provide strong evidence that chemical crosslinking between PEI and PEGDGE occurred as depicted in Scheme S2.

(A)

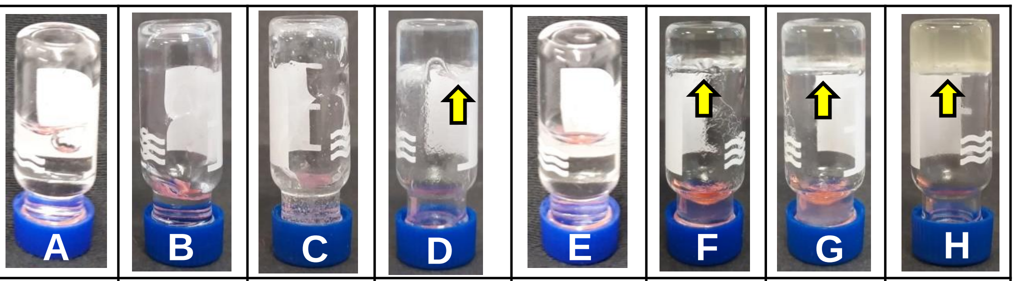

**State**	Fluid	Fluid	Fluid	Gel	Fluid	Gel	Gel	Gel
$\frac{\boldsymbol{n(epoxide)}}{\boldsymbol{n(1^{o}amine)}}$	0	0.23	0.39	0.87	0.98	2.6	4.7	27
**P6.0 / wt%**	0	1.2	2.0	4.2	1.8	11	17	37
**PEI + P6.0 / wt%**	55	53	51	49	21	53	52	51

(B)

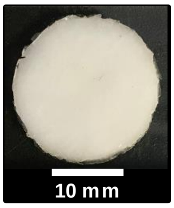

Figure S5. (A) Photographs of PEI/P6.0 mixtures with different primary amine/epoxide ratios and total concentrations. Gels can be seen at the top of the vials for D, F, G and H (arrows). The third row shows the estimated molar ratio of epoxide to primary amine groups. The fourth row shows the P6.0 concentration used. The red colour near the vial necks is a spurious reflection. **(B)** An image of the PEI-(P6.0)$_{0.9}$-NG gel is shown for comparison.

FTIR spectra of the freeze-dried PEI-P6.0 gels from Figure S5 prepared using P6.0 concentrations of 4.2% (Vial D, PEI-$(P6.0)_{4.2}$ gel) and 37% (Vial H, PEI-$(P6.0)_{37}$ gel) were measured and compared to that of PEI and P6.0 (see Figure S6). Unfortunately, the epoxide band at 915 cm^{-1} (red arrow) is too weak in the P6.0 spectrum to allow detection and so the disappearance of such a band cannot be ascertained by FTIR for the gels. The PEI-$(P6.0)_{4.2}$ gel is considered the closest to the covalently bonded complex coacervate PEI-$(P6.0)_{0.9}$-NG system bearing in mind the local concentration of PEI (and P6.0) that occurs during the coacervation process. The FTIR spectral bands for PEI-$(P6.0)_{4.2}$ are in the same positions as those for the parent PEI and P6.0 components. We therefore propose that the only difference experienced by these two components upon forming the PEI-$(P6.0)_{0.9}$-NG gel is covalent crosslinking according to Scheme S2.

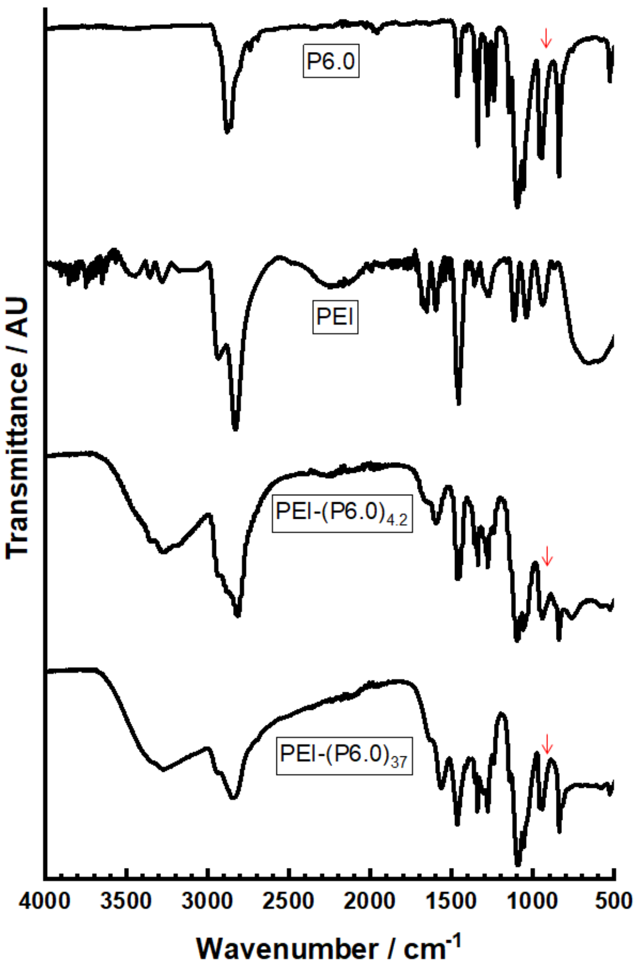

Figure S6. FTIR spectra of P6.0, PEI and freeze-dried Gel D and Gel H from Figure S5. The position expected for the epoxide absorption (915 cm^{-1}) is indicated with a red arrow.

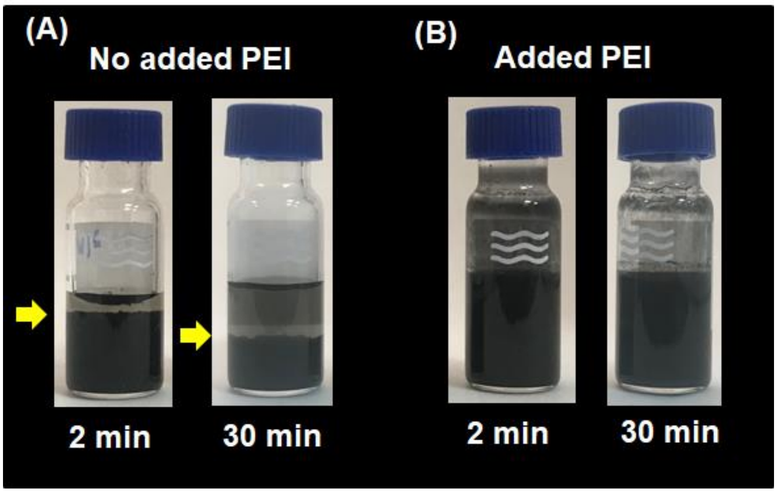

Figure S7. Stability of Gnp dispersion **(A)** without added PEI (left) and **(B)** with added PEI. The photographs were taken 2 or 30 min after mixing. The concentration of Gnp is identical to that used to prepare PEI-(P6.0)$_{0.9}$-NG/Gnp$_{3.1}$ gel. The yellow arrows show the height of the sediment layers. No sediment layers were evident when PEI was added.

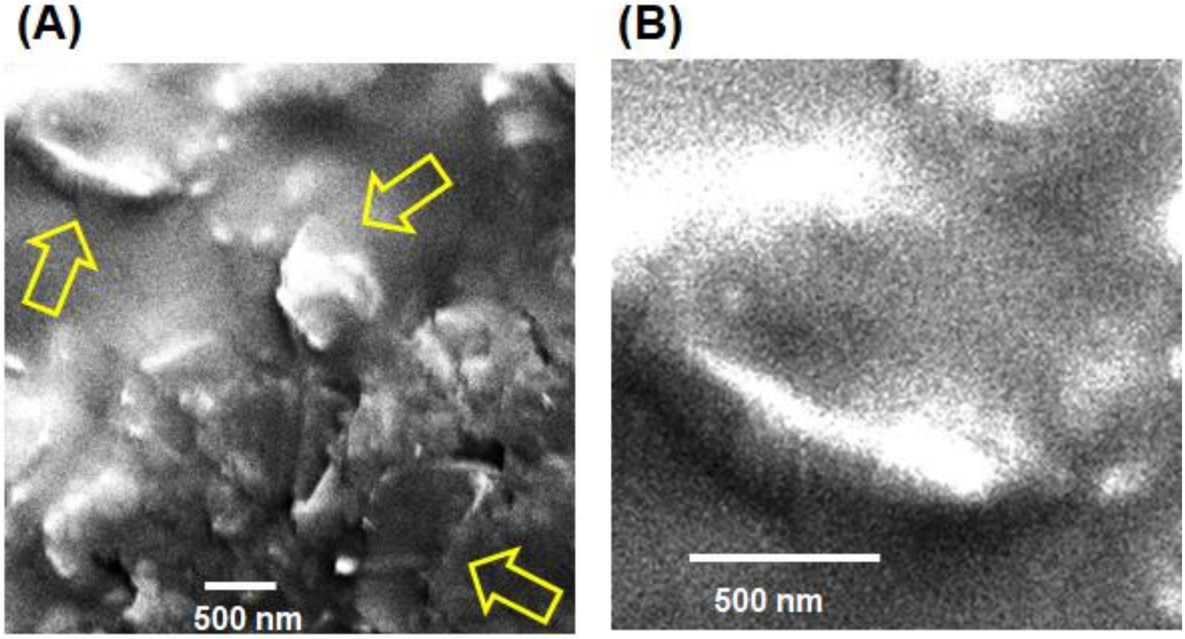

Figure S8 (A) SEM of deposited Gnp particles at deposited from a PEI/Gnp dispersion. An expanded view of partially buried Gnp is shown in **(B)**. The yellow arrows in (A) highlight Gnps.

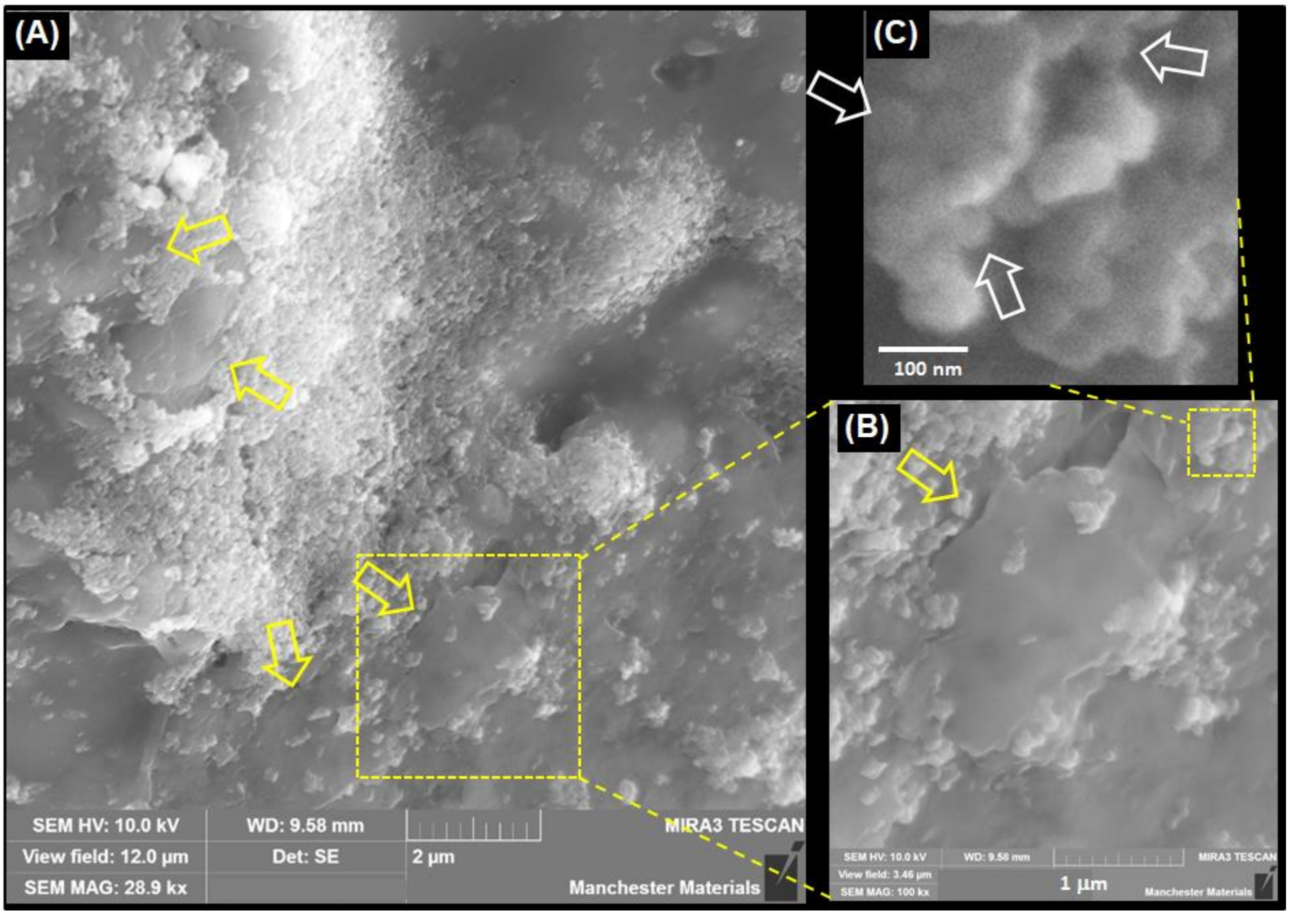

Figure S9. SEM images for PEI-(P6.0)$_{0.9}$-NG/Gnp$_{3.1}$ gel at different magnifications. Gnps are highlighted by the yellow arrows. NGs are highlighted by the white arrows.

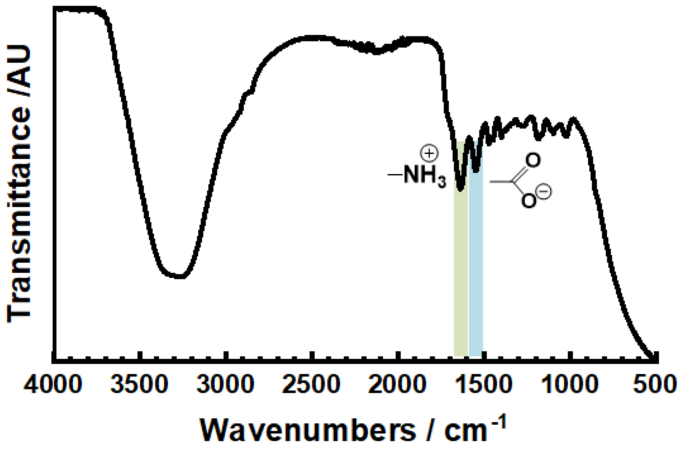

Figure S10. FTIR spectrum for the freeze-dried PEI-(P6.0)$_{0.9}$-NG/Gnp$_{3.1}$ gel. Bands due to ionic groups are shown.

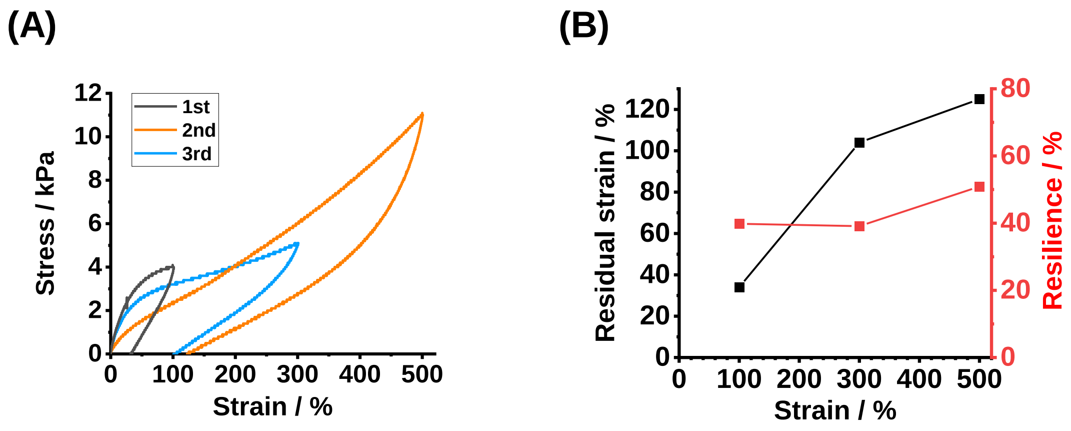

Figure S11. Cyclic tensile data **(A)** and residual strain and resilience **(B)** for PEI-(P6.0)$_{0.9}$-NG/Gnp$_{3.1}$.

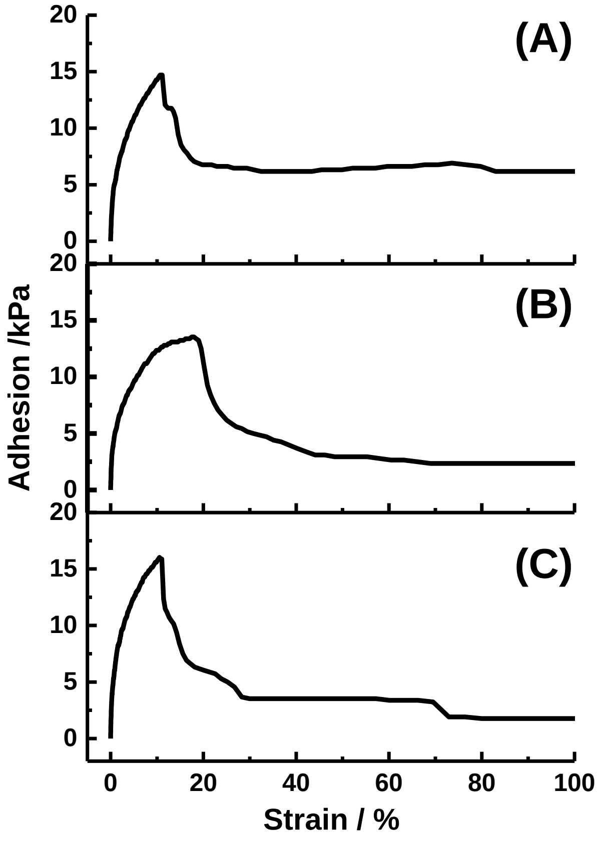

Figure S12. Adhesion versus strain curves for (A) PEI-NG, (B) PEI-(P6.0)$_{0.9}$-NG gel and (C) PEI-(P6.0)$_{0.9}$-NG/Gnp$_{3.1}$ gels adhered to PMMA plates.

NG/Gnp3.1 gel.

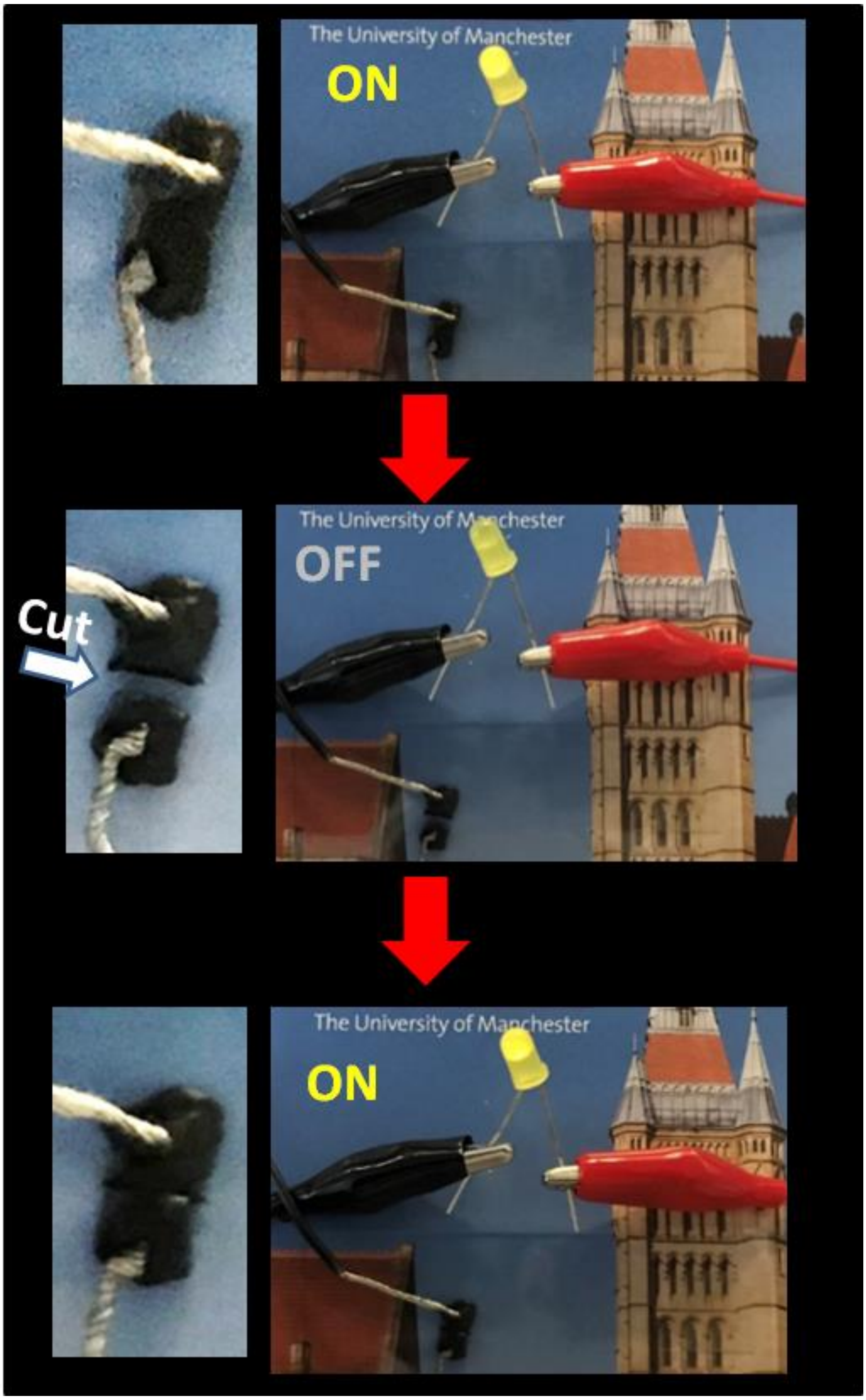

Figure S13. Demonstration of a self-healable electronic circuit with a LED light for PEI-$(P6.0)_{0.9}$-NG/$Gnp_{3.1}$ gel.

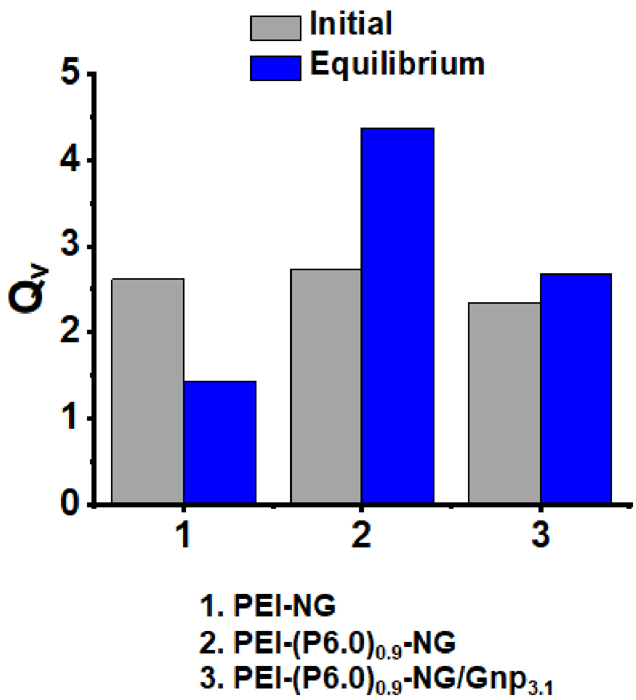

Figure S14. Comparison of the initial and equilibrium volume swelling ratios for the gels. The initial swelling ratio corresponds to the as-made state. The pH values of the as-made gels were 8.6 to 9.8. The equilibrium swelling values were obtained after immersion of the gels in PBS buffer (pH 7.4) for 5 days.

Supplementary table

Table S1. Summary of the SAXS data

Sample	*Physical form*	*q (Å^{-1})*	*Centre-to-centre distance (nm)*	*SAXS pattern characteristic*
NG (20 wt%)	Aqueous dispersion	0.0120	52	Highly ordered
PEI-NG	Gel	0.0100 - 0.0120	52 – 63	Ordered
PEI-$(P0.5)_{4.4}$-NG	Gel	0.0115	55	Ordered
PEI-$(P6.0)_{0.9}$-NG	Gel	0.0100	63	Less ordered
PEI-$(P6.0)_{0.9}$-NG/$Gnp_{3.1}$	Gel	_	_	Porod component

References

1. Cui, Z.; Wang, W.; Obeng, M.; Chen, M.; Wu, S.; Kinloch, I.; Saunders, B. R., Using intra-microgel crosslinking to control the mechanical properties of doubly crosslinked microgels. *Soft Matter* **2016,** *12*, 6985-6994.

2. Kryuchkov, Y. N., Concentration Dependence of the Mean Interparticle Distance in Disperse Systems. *Refract. Ind. Ceram.* **2001,** *42*, 390-392.

3. Wu, S.; Zhu, M.; Lu, D.; Milani, A. H.; Lian, Q.; Fielding, L. A.; Saunders, B. R.; Derry, M. J.; Armes, S. P.; Adlam, D.; Hoyland, J. A., Self-curing super-stretchable polymer/microgel complex coacervate gels without covalent bond formation. *Chem. Sci.* **2019,** *10*, 8832-8839.

4. von Harpe, A.; Petersen, H.; Li, Y.; Kissel, T., Characterization of commercially available and synthesized polyethylenimines for gene delivery. *J. Controlled Release* **2000,** *69*, 309-322.
